# Fabrication of Antibacterial and Functional Films from Starch-Polyvinylpyrrolidone Composite Using Plasma Treatment and Silver Nanoparticles

**DOI:** 10.1021/acsomega.5c03293

**Published:** 2025-08-13

**Authors:** Muhammad Nazrul Islam, Nikitha Modupalli, Md Mahfuzur Rahman

**Affiliations:** Department of Food Science, 3341 University of Arkansas, Fayetteville, Arkansas 72704, United States

## Abstract

Biopolymers and water-soluble nontoxic synthetic polymer composites using silver nanoparticles are astute approaches for antibacterial film fabrication. Moreover, surface treatment of the biopolymeric composite film by cold plasma can enhance the biocidal activity. Silver nanoparticles were synthesized by using the reduction method. Films were fabricated with different ratios of rice starch and polyvinylpyrrolidones (PVP) (1:0, 1:1, and 3:1), with and without silver (Ag) nanoparticles. A plasma jet was used to treat the film’s surfaces by placing the Film 0.5 cm below the plasma discharge. Surface morphology was monitored by scanning electron microscopy (SEM), and the existence of Ag nanoparticles in the film was confirmed by X-ray diffraction (XRD). The UV–vis spectrum at 420 nm confirms Ag nanoparticles, which have an average hydrodynamic radius of 207.3 ± 21 nm, measured by a Zetasizer, and an average particle size of 69.85 ± 2.13 nm, analyzed by transmission electron microscopy (TEM). Moisture content, water absorption, swelling properties, tensile strength, contact angle, and DSC and TGA of all films were studied. It was observed that the moisture content, moisture absorption, and tensile strength increased after the addition of PVP, with few exceptions. All these properties were improved in plasma-treated films. Crystallinity, thermal stability, and glass transition temperature (*T*_g_) were also enhanced when the surface of the films was treated with cold plasma. The antibacterial activity of these films was evaluated by using the agar diffusion method, and silver nanoparticle-containing films showed good antibacterial properties, which increased significantly after plasma jet treatment of the films. The findings indicated that the plasma surface-treated silver nanoparticle-incorporated rice starch–PVP composite film has the potential to be used as an antibacterial film. These films can be used as bandages for wound healing and antibacterial packaging.

## Graphical abstract

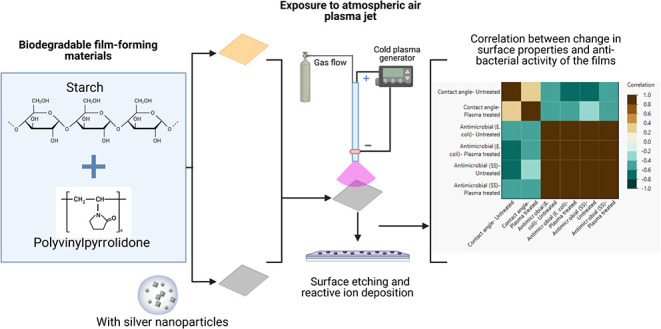

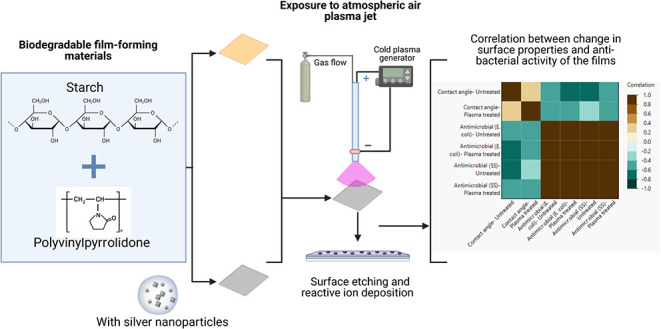

## 1 Introduction

Biopolymer-based antibacterial films have drawn significant attention in both the food packaging and medicine industries due to their versatile properties, including biodegradability, biocompatibility, antimicrobial activity, and sustainability.^1,2^ These films have been widely used as food packaging materials to prolong shelf life, maintain taste and aroma, and reduce spoilage caused by microbial contamination.^3^ In medicine, biopolymer-based thin and elastic films are used for wound healing.^4,5^ They protect wounds from bacteria while allowing oxygen and moisture to pass through. They are often used as bandages or wound dressings to treat superficial wounds and minor burns and can be shaped to fit different body parts.^6^

Rice starch is a polymeric mixture of two different types of α-glucans, such as amylose and amylopectin.^7^ Although branching might occur on the amylose chains, it is considered a linear polymer of α-1,4-linked glucose molecules. However, amylopectin possesses a branched structure with α-1,4-linked glucose molecules and about 5–6% α-1,6-linkages at the branch points.^8^ Glass transition, swelling, and gelatinization of starch depend on the amylose-to-amylopectin ratio, and water acts as a plasticizer for the amorphous regions of the starch granule.^9^ Film formation characteristics of rice starch also depend on the amylose and amylopectin ratio, structure, chain length, types of plasticizers, temperature, and various processing conditions.^10,11^ Overall, rice starch is an inexpensive, biodegradable, nontoxic, semicrystalline biopolymer mostly used in food, packaging, and bioplastic materials.^12^ However, these types of biopolymers are moisture-sensitive, brittle, and have weak thermal and mechanical properties.^13^ To enhance the mechanical properties of biopolymers, a composite of synthetic and biopolymers is popular for maintaining a balance between processability and suitability. Polyvinylpyrrolidone (PVP) can be a good choice for preparing rice starch composite, which is a water-soluble synthetic polymer prepared by the radical polymerization of the *N*-vinylpyrrolidone monomer with a suitable initiator. Though most synthetic polymers^14^ and antibacterial polymeric agents^15,16^ are difficult to synthesize, water-insoluble, nonbiodegradable, and toxic, PVP is an inert, nontoxic, temperature-resistant, pH-stable, biocompatible, biodegradable polymer with high chemical and thermal resistance and film-forming properties.^17^ The use of silver (Ag) nanoparticles as an antimicrobial agent has been well-known for centuries, and Ag nanoparticle incorporation in composite films still plays an important role in various medical and healthcare systems.^18^ Ag nanoparticles adhere to the surface of the bacterial cell wall and penetrate the cell membrane to damage the intracellular structure. Cellular toxicity occurs in the presence of Ag nanoparticles and creates oxidative stress on the cell wall of the bacteria when DNA loses its replication ability.^19,20^ Moreover, cold plasma etching of nanostructured surfaces in the presence of silver enhances antimicrobial performance.^21^

Cold plasma is a green and environmentally friendly method that does not involve chemical activity and can be used at atmospheric pressure and temperature.^22^ Plasma discharge high-energy electrons collide with the surface of the films to form active species, ions, atoms, and radicals through complex processes such as excitation, dissociation, and ionization.^23^ Plasma jets are generated in a dielectric tube with a high flow of air or a noble gas through the tube. The plasma jet plume varies with the applied voltage, frequency of power, type of gases, flow rate, plasma generation time, and distance between the film’s surface and the nozzle. Plasma jets have demonstrated potential for various applications, such as chemical and structural modification of macromolecules, degradation of large molecules, surface etching, and depolymerization.^24^ Cold plasma-generated oxygen species deposit on the surface of the film, which changes the topology of the film, making it difficult for bacteria to attach to this rough surface. Plasma-generated oxygen species can oxidize Ag nanoparticles, which also play a role in enhancing antibacterial activities. Moreover, plasma-generated oxygen species can damage the bacterial cell membrane and disrupt reproductive activity. Therefore, plasma jet-treated Ag-incorporated rice starch–PVP composite films are used in the biomedical field applications, such as antibacterial agents, disinfectants, antiseptics, and wound healing promoters.^25,26^

Though many articles have been published on various sources of starch film, work on rice starch film is very limited, and very few works have been reported on the rice starch–PVP composite film. Moreover, the incorporation of Ag nanoparticles with rice starch–PVP composite film is a unique idea for creating an antibacterial film. The novelty of this work lies in the modification of film properties and the enhancement of the antibacterial activity of the Ag nanoparticle-induced rice starch–PVP composite film through cold plasma. This study aims to fabricate functional films using rice starch and rice starch–PVP nanocomposite films that can be used in wound healing. The objectives of this study are to (i) fabricate a biodegradable film using rice starch and rice starch–PVP nanocomposite, (ii) use Ag nanoparticles to induce antimicrobial activity in the films, and (iii) modify their surface using cold plasma to enhance the functional properties of the film. The study is expected to reveal the effects of PVP, Ag nanoparticles, and plasma species on rice starch film, which would contribute to knowledge for further studies on the antibacterial activities of nanocomposite films.

## 2 Materials and Methods

### 2.1 Materials

Silver nitrate (AgNO_3_) (CAS No. 7761–88–8) and glycerol (CAS No. 56–81–5) (purity 99.5%) were purchased from VWR-BOH Chemicals, USA. Sodium citrate dihydrate (CAS No. 6132–04–3) was purchased from Avantor, USA, and starch was purchased from MP Biomedicals, USA. For the preparation of phosphate buffer, dipotassium hydrogen phosphate (K_2_HPO_4_) (CAS No. 7758–11–4) and potassium dihydrogen phosphate (KH_2_PO_4_) (CAS No. 7778–77–0) were purchased from Ataman Kimya, Turkey. 1-Vinyl-2-pyrrolidone (VP) (CAS No. 88–12–0) was also obtained from Ataman Kimya, Turkey. All of the chemicals were used as received without further purification. PVP was synthesized using the free radical polymerization method using H_2_O_2_ as an initiator.^27^ Silicon molds were purchased from Amazon for casting the film.

### 2.2 Synthesis of Silver Nanoparticles

The silver nanoparticles were prepared from an AgNO_3_ solution by using sodium citrate as a reducing agent.^28^ For the synthesis of silver nanoparticles, 180 mg of AgNO_3_ was dissolved in 500 mL of distilled water in a 1 L round-bottom flask and heated to 100 °C under a reflux condenser. Twenty mL of trisodium citrate solution (1% w/v) was added to the boiling AgNO_3_ solution with magnetic stirring, and the mixture was boiled and stirred continuously for 1 h. The Ag-sol prepared was greenish-yellow. The Ag-sol was condensed and purified by centrifuging at 8000 rpm for 30 min.

### 2.3 Synthesis of Polyvinylpyrrolidone (PVP)

Polyvinylpyrrolidone (PVP) was synthesized in buffer solutions using H_2_O_2_ as the initiator, while controlling the monomer-to-initiator ratio by following our previous article.^27^ A 0.2 M buffer solution at pH 7.58 was prepared by dissolving 3.57 g of K_2_HPO_4_ and 0.624 g KH_2_PO_4_ in 96 mL of water with continuous stirring. 56 mL of vinyl pyrrolidone was dissolved in the buffer solution and heated to 86 °C for 30 min. To synthesize PVP, 0.35 mL of H_2_O_2_ was then added to the monomer solution and continuously stirred for 6 h at 86 °C.^27^

### 2.4 Preparation of Polyvinylpyrrolidone (PVP)–Starch Film through the Pour Casting Method

To prepare the starch–PVP composite film, a desired amount of PVP (0.75 g for 1:1 and 0.37 g for 3:1) was dissolved in 25 mL of water at room temperature. Starch was then added to the PVP solution and heated at 85 °C for 15 min. Glycerol (0.45 g) was added to the PVP–starch solution, and heating was continued for approximately 1 h to allow gel formation.^29^ The gel was degassed for 5 min in an ultrasound bath, poured into a silicone mold for casting, and dried for over 48 h under continuous airflow in a fume hood at room temperature.

### 2.5 Surface Treatment of the Film by Plasma

Plasma was produced by a plasma generator (Leap 100, Plasma Leap Technologies, Sydney, Australia) equipped with a transformer, and a jet discharge reactor was used to generate plasma by using air as the source gas. The generator was operated at a duty cycle of 87 μs, an input voltage of 150 V, and a discharge frequency of 1500 Hz. The surface of each film was treated under the jet for 30 min.

### 2.6 Characterization by Fourier Transform Infrared Spectroscopy (FTIR)

FTIR spectra were collected using an Agilent Cary 630 FTIR spectrometer, equipped with an attenuated total reflectance (ATR) accessory containing a potassium bromide crystal. FTIR data acquisition was conducted with wavenumber ranges 650 cm^–1^ to 4000 cm^–1^, at a 4 cm^–1^ spectral range and 64 scans for precision at room temperature. A background scan was performed for each sample to nullify the effect of the background atmosphere on data acquisition.

### 2.7 Ultraviolet–Visible Spectroscopy (UV–Vis Spectroscopy)

The absorbance of the Ag nanoparticle was investigated using a UV–vis spectrophotometer (Shimadzu UV-1900i, Japan). UV–vis spectra were observed at 250–700 nm using a quartz cuvette.

### 2.8 Particle Size Determination

The hydrodynamic diameter of the silver nanoparticles was determined using a Zetasizer instrument (Anton Paar Litesizer 500, USA). The silver nanoparticles were dispersed in water, and data were recorded at 25 °C. The particle size was determined by an FEI Titan 80–300 HRTEM (High-Resolution Transmission Electron Microscope) equipped with a field emission gun, operated at 10 kV and 150000× magnification.

### 2.9 Contact Angle Measurements

The contact angle was measured using a static/dynamic contact angle tensiometer (SEO model, Phoenix 300, Kromtech Alliance Corp., London, U.K.). The static contact angles (θ_est_) were determined according to the Young–Laplace equation by observing sessile drops of ultrapure water (4 μL) deposited on the surface of the films under air relative humidity of 67 ± 5% and a temperature of 25 ± 2 °C. Each reading was taken in triplicate for precision.

### 2.10 Tensile Strength Measurements

Film strips with accurate measurements of 40 × 10 mm were fixed into the texture analyzer’s film extension grips (initial grip separation of 20 mm). The texture analyzer pulled them apart at a rate of 2 mm/s for 20 mm. The TS was calculated using the following eq 1:

1 $$\mathrm{Tensile Strength}=\frac{\mathrm{Peak Load}\;(N)}{\mathrm{Cross-Sectional Area}\;(mm^{2})}$$

### 2.11 X-Ray Diffraction Analysis (XRD)

The structural properties of the films were characterized by X-ray diffraction (XRD) analysis using a Philips X’Pert MPD (Almelo, Netherlands) Double System Diffractometer equipped with a Ge monochromator in the Bragg–Brentano reflection geometry. The step size was 0.02°, and the time per step was 1 s. The analysis was performed in a broad range of 2θ angles (5 to 80°) at a voltage of 45 kV and a current of 40 mA. The wavelength of the X-ray was 1.5406 Å.

The crystallinity index of the films was calculated using a previously proposed modification of the Nara and Komiya method.^30^ A smoothed curve connecting the peak baselines was superimposed onto the recorded XRD pattern. The area above the smoothed curve corresponded to the crystalline portion of the film, while the area under the curve corresponded to the amorphous region of the film. The crystallinity index was calculated as the ratio between the area of the crystalline phase and the total area under the XRD curve using the eq 2:

2 $$\mathrm{Relative Crystallinity}=\frac{\mathrm{Crystalline area}}{\mathrm{Crystalline area}+\mathrm{Amorphous area}}$$

### 2.12 Thermogravimetric Analysis (TGA)

Thermogravimetric analysis was performed using a Q50 instrument (TA Instruments, USA). The samples (approximately 15.5 ± 0.1 mg) were placed in a standard platinum pan. The scan was run at 10 °C/min under a nitrogen flow. The mass change was measured from 25 to 400 °C. Samples were stabilized at room temperature and 50% humidity in a desiccator before analysis.

### 2.13 Differential Scanning Calorimetry (DSC) Analysis

The glass transition temperature of the films was analyzed by using a Q2000 differential scanning calorimeter (TA Instruments, USA). The scan was run at 10 °C/min under a nitrogen flow, ranging from 100 to 250 °C. Samples were stored at room temperature and 50% humidity in a desiccator before analysis

### 2.14 Water Uptake Capacity of the Films

The film was cut into a 1 ×1 cm square and dried for 24 h at 105 °C. The sample was weighed, and the films were placed in a desiccator for 24 h. The samples were properly weighed again by an electric balance. The film’s water uptake capacity was calculated by using the following eq 3:

3 $$\mathrm{Water Uptake}\%=\frac{W_{s}-W_{d}}{W_{d}}$$

where *W_s_* and *W_d_* stand for the weights of the oven-dried films and the weights of the swollen films at 24 h, respectively. The analysis was done in duplicate for both groups.

### 2.15 Swelling Profiles of the Films

The swelling test was used to understand the swelling behavior of the films.^31^ The film was cut into a 1 ×1 cm square size and dried for 24 h at 105 °C. Distilled water was used as the solvent for the swelling test. The swelling ratio of the composite films was determined using the following eq 4:

4 $$\mathrm{Swelling Rate}\;\%=\frac{W_{s}-W_{d}}{W_{d}}$$

The weight of the swollen film is denoted by *W_s_*, and the dry film’s weight is denoted by *W_d_*.

### 2.16 Antimicrobial Activity Test

Two different types of pathogenic bacteria (Gram-positive and Gram-negative) were used to test the antimicrobial behaviors of different films. The Gram-positive bacteria *Staphylococcus* *sps.* and Gram-negative bacteria *Escherichia coli* were used as representative groups. An antibiotic drug, 100 μg/mL rifampicin, was used as a positive control, while distilled water was used as a negative control. The disc-agar diffusion method was used to investigate the antibacterial activities of each of the 12 films. To establish the method, we ran several rounds of initial cultures. Sterilized LB agar (for *E. coli*) and blood agar (for *Staphylococcus* *sps.*) were poured into sterile Petri dishes and allowed to solidify. The agar plates were then inoculated with 100 μL of fresh culture containing at least 1.0 × 10^6^ CFU/mL of each bacterial strain separately. The inoculated plates were incubated at 37 °C for 24 h to grow the bacterial cultures. After 24 h, wells of 1 cm diameter were bored into the inoculated agar. One cm diameter film samples were placed in the wells, and the plates were again incubated. The bottom of the agar wells was sealed with an agarose solution to avoid cross-contamination. The plates were incubated overnight at 37 °C. The zones of inhibition (ZOI) were visually measured by using a vernier caliper. The plates were incubated at 30 °C for 3 days, and ZOI was quantified after every 24 h. Following the incubation phase, the ZOI was quantified.^32^

### 2.17 Statistical Analyses

The statistical analysis of the data was performed through analysis of variance (ANOVA) using the JMP statistical program (JMP Statistical Discovery LLC, NC, USA). Each experimental evaluation was performed in three replicates, and the results were reported as the average± standard error. The differences between means were evaluated using Tukey’s multiple range test (*p* < 0.05). The data were expressed as the mean ±SD (standard deviation). Additionally, principal component analysis and correlation analysis were performed to understand the interaction of the film composition with the properties it exerted.^33^

## 3 Result and Discussion

### 3.1 Synthesis of Silver Nanoparticles and Fabrication of Films

#### 3.1.1 Synthesis of Silver Nanoparticles

The silver nanoparticles were prepared as a nanosilver sol, or a silver colloid, by reducing AgNO_3_ with sodium citrate, and successful synthesis was confirmed by UV–vis absorbance. Figure 1 shows the UV–vis absorbance of the Ag nanoparticles. The UV–vis absorbance of silver nanoparticles showed a peak at 425 nm. Both the UV–vis peak and the color change during the reaction (Figure S1) represent the successful synthesis of Ag nanoparticles. Singh et al. also reported a UV–vis spectrum for Ag nanoparticles in a similar region.^34^ The hydrodynamic diameter of the nanoparticles in the water solution is 207.3 ± 21 nm, and the polydispersity index is 23.3 ± 2.6%. The particle size distribution of Ag nanoparticles in water is shown in Figure S3. The hydrodynamic radius of the particles is larger due to surface water molecules and partial aggregation in the solution during centrifugation.^35,36^ To confirm the effective particle size of Ag nanoparticles, the transmission electron microscope (TEM) image of the particles was analyzed, and the average particle size was found to be 69.85 ±2.13 nm (Figure 1b). The particles appeared close to each other in the micrograph and were not significantly aggregated. The shape of the synthesized Ag nanoparticles was primarily spherical, while some appeared slightly rod-shaped in the micrographs. The size of the Ag nanoparticles depends on various factors, such as the reduction capacity of citrate, the pH of the solution, the temperature, and the concentration of the solution.^37,38^

**Figure 1:**
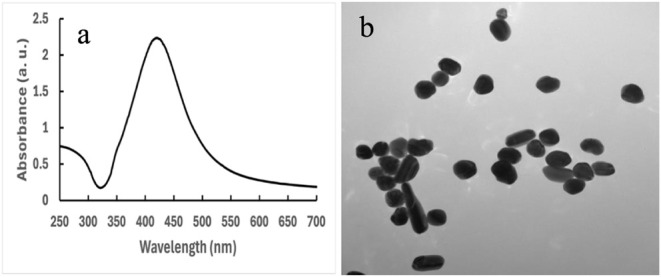
(a) UV–vis of silver nanoparticles. (b) Transmission electron microscope (TEM) image of silver nanoparticles.

#### 3.1.2 Fabrication of Film and Surface Treatment by Plasma Jet

A total of 6 films were fabricated, and the composition of the film ingredients is shown in Table S1. Films 1, 2, and 3 are composed of 100%, 50%, and 75% starch, respectively, with the remaining portion being PVP. Films 4, 5, and 6 were prepared using the same starch-to-PVP ratios as Films 1, 2, and 3, but were fabricated with 1% silver nanoparticles. All films underwent plasma treatment and were denoted as 1P, 2P, 3P, 4P, 5P, and 6P, respectively. Only the starch film is white, while the starch:PVP (1:1) film is yellowish; however, the starch (3:1) looks less yellowish, with a ratio of PVP lower than the preceding one (Figure 2). Ag nanoparticle-containing rice starch and rice starch–PVP films (Films 4–6) look gray in color.

**Figure 2:**
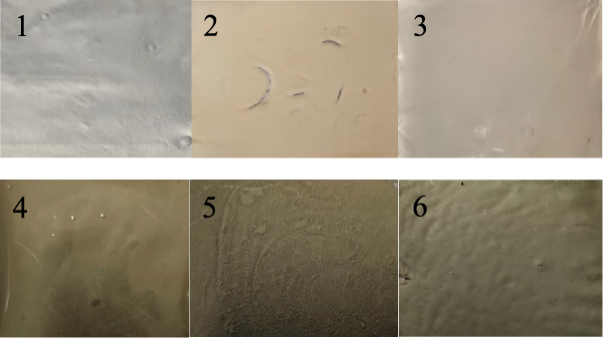
Picture of prepared films: Film 1: 100% starch, Film 2: 50% starch and 50% PVP, Film 3: 75% starch and 25% PVP, Film 4: 100% starch with 1% Ag nanoparticles, Film 5: 50% starch and 50% PVP with 1% Ag nanoparticles, Film 6: 75% starch and 25% PVP with 1% Ag nanoparticles.

### 3.2 Characterization of Films

#### 3.2.1 Fourier Transform Infrared Spectroscopy (FTIR) of Films

FTIR spectroscopy was used to analyze the structural properties and identify the inclusion or deletion of any specific functional groups in the films. In the FTIR spectra, 3207 cm^–1^ indicates the O–H bond, and 993 cm^–1^ represents the C–O bond of starch (Figure S4). The spectra at 2924 cm^–1^ are for the C–H bond of sp^3^ hybridized carbon. In PVP, FTIR spectra bands at 2926 cm^–1^ and 1446 cm^–1^ also correspond to the C–H bond from the CH_2_ group. The peak located at 1649 cm^–1^ is for the stretching vibration of the C=O bond in the pyrrolidone group, and the absorption band at 1286 cm^–1^ is for the C–N bond’s bending vibration due to the pyrrolidone structure.^39^ The film (Film 2) made of starch and PVP (1:1) represents both starch and PVP in the FTIR spectra, where 3287 cm^–1^ indicates the O–H bond, 1025 cm^–1^ represents the C–O bond of starch, band 1649 cm^–1^ is for the C=O stretching vibration, and the absorption band at 1291 cm^–1^ is for the C–N bond’s bending vibration in the pyrrolidone group. The spectra at 2929 cm^–1^ and 1448 cm^–1^ are for the C–H of CH_2_ of the polymer chain in the film. A similar pattern was observed in the FTIR of Films 1 and 2, as shown in Figure S5 . The only difference is the intensity of the O–H bond peak at 3207 cm^–1^ because of the variation in the ratios of starch and PVP used in the films. The −OH peak at 3270 cm^–1^ is more intense in Film 1 than in Film 2, as it is made of only starch that contains hydroxyl groups from amylose and amylopectin. Both Films 2 and 3 possess rice starch and PVP, but Film 2 contains a higher percentage of PVP, which shows a highly intense C=O stretching vibration at 1647 cm^–1^ of the pyrrolidone group. Films 4, 5, and 6 were prepared using an Ag nanoparticle sol, and their FTIR spectra are shown in Figure S6. No significant differences were observed in the FTIR of films prepared by using the Ag nanoparticle sol, as the ratio of starch and PVP remained the same.

FTIR spectra of plasma-treated films are shown in Figures S7–S11, with a representative Film 5 is shown in Figure 3. After plasma treatment, the peak intensities of all peaks decreased, but not their positions at different frequencies.^40^ The −OH peak (3281 cm^–1^) intensity decreased due to cross-linking of starch and formation of C–O–C (995 cm^–1^) by plasma treatment in all films.^41^ In the pyrrolidone group, the C–H bond peak intensity at 3281 cm^–1^, the C=O bond peak intensity at 1647 cm^–1^, and the C–N bond bending vibration at 1291 cm^–1^ are also decreased. The plasma treatment might cause the rupture of C–C and C–H bonds in the PVP chain, while the C =O group forms hydrogen bonds with starch.^42,43^

**Figure 3:**
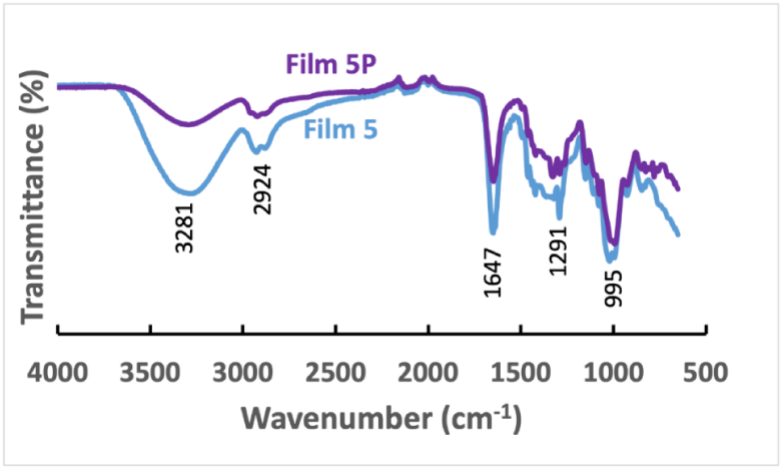
FTIR of the film before plasma, Film 5, and after plasma treatment, 5P.

#### 3.2.2 x-Ray Diffraction (XRD) Characterization of Films

The X-ray diffraction (XRD) patterns of films from rice starch and PVP composites are shown in Figure 4. The rice starch film (Film 1) exhibited a singlet at approximately 2θ = 17.5°, a doublet at 2θ = 19.75° and 20.25°, and a singlet at 2θ = 22°. However, after plasma treatment, the film (Film 1P) exhibited a singlet at approximately 2θ = 18°, another singlet at 2θ = 20°, and a singlet at 2θ = 22°. The peak area significantly increased after plasma treatment, representing an increase in the degree of crystallinity from 17.94% to 20.94% (Table 1). The increase in crystallinity indicates the change in orientation of straight-chain starch composed of double helices inside the crystal.^41^ A possible explanation for this is that plasma treatment can enhance cross-linking inside the starch film and change the internal structure of starch between crystalline and amorphous states. Chen et al. reported that plasma can damage the amorphous region more severely than the crystalline region due to surface penetration of only a few nanometers.^41^ After the addition of 50% PVP with rice starch (Film 1), the degree of crystallinity increased from 17.94% to 19.11%. The crystallinity increases in the starch–PVP composite due to hydrogen bonding with the OH of amylose and the C=O of PVP, as well as bonding between amylopectin and PVP.^44^ Plasma surface treatment further increased crystallinity to 32.33% due to internal cross-linking of polymer chains.^45^

**Figure 4:**
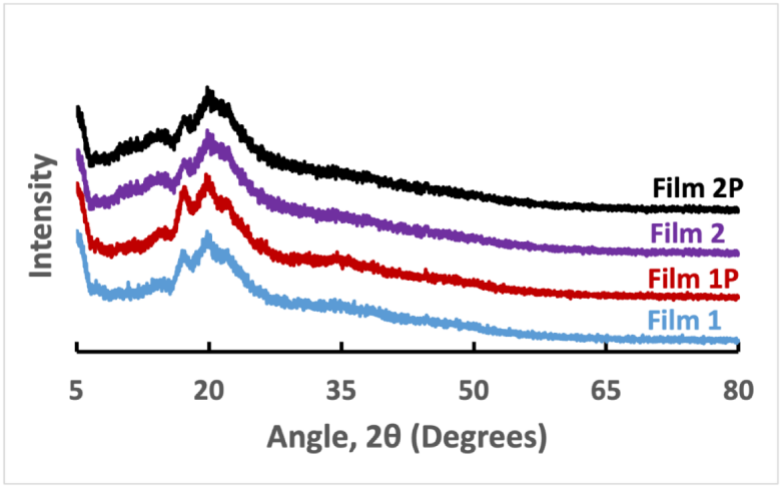
XRD of films: Film 1: 100% starch; Film 1P: Film 1 treated by plasma; Film 2: 50% starch and 50% PVP; Film 2P: Film 2 treated by plasma.

**Table 1:** Degree of Crystallinity of the Films

	Before Plasma Treatment		After Plasma Treatment	
Films	Film Number	Crystallinity %	Film Number	Crystallinity %
Starch	1	17.94	1P	20.94
Starch–PVP (1:1)	2	19.11	2P	32.33
Starch–PVP (3:1)	3	22.05	3P	24.12
Ag–Starch	4	82.87	4P	83.87
Starch–PVP (1:1)–Ag	5	93.99	5P	93.53
Starch–PVP (3:1)–Ag	6	92.11	6P	92.18

XRD patterns and the degree of crystallinity of plasma-treated films are shown in Figure 5 (details in Figures S12–S15) and Table 1. Film 6 showed a low-intensity broad peak at a 2θ of 20° for starch and PVP in the film. However, high-intensity sharp peaks were observed at 2θ values of 38.3°, 44.5°, 65.5°, and 77.4°, which are characteristic peaks of Ag nanoparticles reflecting Bragg–Brentano geometry at planes 110, 200, 220, and 311, respectively.^46^ The degree of crystallinity of this film increased from 92.11% to 92.18%, and the total peak area slightly increased after plasma treatment. Although 100% starch film has a much lower crystallinity than the Ag nanoparticle-incorporated film, the crystallinity enhancement rate is higher in the starch film after cold plasma treatment (Table 1). This might be due to the plasma-induced higher cross-linking ability in the starch films compared to Ag nanoparticle-incorporated starch films.^47^ Moreover, Khatib et al. reported that plasma treatment of polyetheretherketone resulted in an increase in the global degree of crystallinity from 9.5% to 14.9%.^48^ After the addition of Ag nanoparticles, the crystallinity of the films increased almost four times. The crystallinity of the films was also increased by the addition of PVP. The crystallinity is more prominent in films with Ag nanoparticles after plasma treatment.

**Figure 5:**
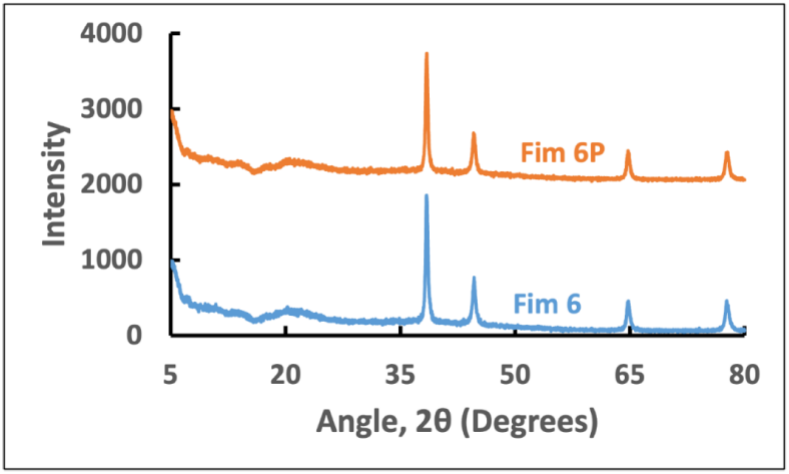
XRD of films: Film 6: 75% starch and 25% PVP with 1% Ag nanoparticles; Film 6P: Film 6 treated by plasma.

#### 3.2.3 Contact Angle (CA)

The surface hydrophobicity and hydrophilicity of films affect wettability, coating ability, adhesion, and frictional properties.^49^ A decrease in contact angle means an increase in the hydrophilicity of the film. The contact angle data are presented in Figure 6, and images are shown in Figure S16. The rice starch film’s contact angle is 75.55 ± 2.96°, and plasma treatment does not show any significant impact on it. Moreover, films composed of 75% of the starch and 25% PVP do not show any significant difference after plasma treatment. However, the addition of 50% PVP to 50% rice starch increased the contact angle from 75.55 ± 2.96° to 100.48 ± 2.35°; however, it decreased to 86.93 ± 2.83° after plasma treatment. A similar result was reported in the literature, indicating that the hydrophilicity of corn starch increased after plasma treatment.^29^ The higher hydrophilicity of plasma-treated films can be attributed to the cross-links formed by plasma-induced oxygen-containing polar and hydrophilic groups (*e.g*., −OH, −CO, and −COOH) on the film surface.^50^ The contact angle of films decreased after 30 s (Figure S17) and followed nearly similar trends to the instant contact angle.

**Figure 6:**
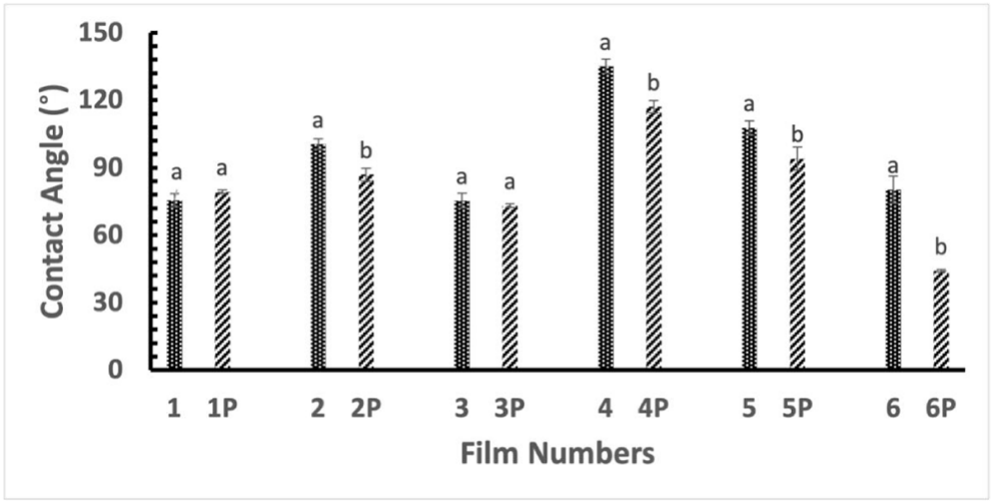
Contact angle measurements of the films. Film 1: starch, Film 2: starch–PVP (1:1), Film 3: starch–PVP (3:1), Film 4: starch–Ag, Film 5: starch–PVP (1:1)–Ag, Film 6: starch–PVP (3:1)–Ag. “P” denotes plasma treatment of the film.

The addition of 1% Ag nanoparticles to the films significantly increased the contact angle, or hydrophobicity, of the starch film from 75.55 ± 2.96° (Film 1) to 135.13 ± 3.08° (Film 4). The Ag nanoparticles reduce the surface energy and increase surface roughness, forming nanohierarchical structures where air is trapped, thereby minimizing the interaction between the film and water droplets.^51,52^ The contact angle in the composite films of 50–50 PVP–starch increased from 100.48 ± 2.35° (Film 4) to 107.73 ± 3.15° (Film 5). This indicates that the enhancement of surface hydrophobicity in Ag nanoparticle-loaded composite films is due to the inclusion of hydrophobic metallic silver in the starch–PVP polymer matrix.^53^

Like other films, plasma treatment reduced the contact angle of all Ag nanoparticles embedded in the films; for example, the contact angle of Film 4 (135.13 ± 3.07°) was reduced to 117.08 ± 2.80 ° in Film 4P after plasma treatment (Figure 6). Plasma-generated reactive species reduced C–H groups and introduced polar oxygen-containing groups, increasing surface polarity and thereby lowering the contact angle in protein films. Overall, Ag nanoparticles enhance the films’ hydrophobicity; however, plasma treatment increases the films’ hydrophilicity.

#### 3.2.4 Thermogravimetric Analysis (TGA)

The thermogravimetric analysis (TGA) was conducted to understand the effect of heat and weight loss on composite films and to find the impact of plasma and Ag nanoparticles on the thermostability of the films. In Figure 7a,b, the weight percent curves with temperature for all six films, before and after plasma treatment, can be divided into three major transitions: (i) free water and structural water evaporate in the range of 25 to 180 °C;^54^ (ii) 180 to 240 °C, when physical decomposition of the films occurs;^55^ and (iii) combustion of the films occurs in the range of 240 to 400 °C.^56^ All of the films, before plasma treatment, lost 4–6% weight from room temperature to 180 °C due to the loss of residual water and absorbed moisture. Except for the rice starch film and the Ag nanoparticle-containing rice starch and PVP (1:1) film, all other films showed similar weight loss patterns during the decomposition stage. The rice starch film’s decomposition began at 274.30 °C and ended at 315 °C with 38.38% weight loss, whereas the rice starch–PVP (1:1) film’s onset decomposition temperature was 224.52 °C, and it ended at 289 °C with 31.21% weight loss. So, the thermal stability of the starch film increased with the addition of PVP, which is also evident from the weight loss of Film 3. A similar result was observed (thermal degradation starting at 251 °C and ending at 313 °C) in propolis nanoparticles incorporated into starch films.^57^ Moreover, the Ag nanoparticle- containing starch–PVP film (Film 5 in Figure 7b) showed the highest thermal stability among the studied films, with 41.01% remaining weight of the film. The Ag ion receives a lone pair of electrons from the ligand of −N and C=O in the pyrrolidone ring, which occupy the s and p orbitals of the silver ion to form a complex compound.^34^ Moreover, amylose and amylopectin in starch might form stable hydrogen bonds with the pyrrolidone ring of PVP, contributing to the highest thermal stability among all films.

**Figure 7:**
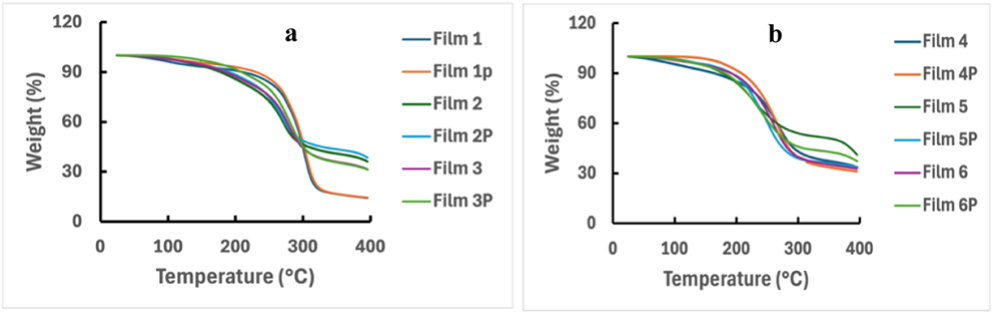
(a) TGA of the films: Film 1: starch; Film 2: starch–PVP (1:1); Film 3: starch–PVP (3:1). (b) TGA of Ag-incorporated films: Film 4: starch–Ag; Film 5: starch–PVP (1:1)–Ag; Film 6: starch–PVP (3:1)–Ag. “P” denotes plasma treatment of the film.

For Films 1 to 3, overall thermal stability was enhanced after plasma treatment (Figure 7a). Though no significant changes were observed in the residue at 395 °C for Films 1 and 3 after plasma treatment, a 2.32% higher residue of Film 2P at 395 °C was found after the plasma treatment of Film 2. Rather than surface modification, the internal cross-linking in the polymers by the plasma treatment plays a crucial role in their relatively high thermal stability.^58^ Physical decomposition was delayed by plasma treatment in Film 4P; however, the plasma-treated Film 5P showed significant changes in the combustion region. The Ag nanoparticle-containing Film 6P showed better thermal stability after plasma treatment (Figure 7b). Rao et al. also showed thermal stability enhancement of PVA-based films after plasma treatment.^58^

#### 3.2.5 Differential Scanning Calorimetry (DSC)

Differential scanning calorimetry (DSC) is a technique for identifying the thermal behavior of materials through the glass transition temperature.^59^ The glass transition of films was measured to understand the effect of PVP, Ag nanoparticles, and plasma on the films. The DSC curves of 100% starch film and *T*_g_ analysis before and after plasma treatment are shown in Figure S18a,b. The *T*_g_ values of all films obtained from the DSC graph are summarized in Table 2. The glass transition temperature (*T*_g_) of 100% rice starch film (Film 1) was found to be 139.51 °C, which decreased to 127.7 °C after the addition of 50% PVP with starch (Film 2). However, the *T*_g_ value increased from 139.51 to 146.19 °C after the addition of 1% Ag nanoparticles. The reduction of the *T*_g_ value of an amorphous and/or semicrystalline polymer and biopolymer reveals a decrease in the intermolecular forces between the polymer chains and their composite, thereby increasing the local chain flexibility by lowering the *T*_g_.^57^ However, after the addition of Ag nanoparticles, the *T*_g_ value increased, which means that the polymer chain flexibility decreased due to the inclusion of Ag nanoparticles. This is because of the electrostatic interaction between Ag nanoparticles and electronegative atoms in the amylose, amylopectin, and PVP composite. For instance, Film 1, prepared from 100% rice starch, and Film 4, which includes the addition of 1% Ag nanoparticles to Film 1, had *T*_g_ values of 139.51 and 146.19 °C, respectively. The literature also reports a similar effect of nanoparticles on starch films.^57,60^

**Table 2:** Glass Transition Temperature (*T*_g_) of the Films

		Before Plasma Treatment			After Plasma Treatment		
Film Types	Film code	Start point (°C)	End point (°C)	*T*_g_ (°C)	Start point (°C)	End point (°C)	*T*_g_ (°C)
RS	1	138.71	140.39	139.51	144.18	144.46	144.31
RS–PVP (1:1)	2	127.63	127.75	127.7	143.04	143.18	143.21
RS–PVP (3:1)	3	154.11	154.57	154.39	153.46	153.67	153.64
Ag–Starch	4	146.09	146.38	146.19	149.52	149.65	149.79
RS–PVP (1:1)–Ag	5	135.44	135.94	135.8	158.65	158.75	158.77
RS–PVP (3:1)–Ag	6	137.76	137.87	137.59	146.09	146.18	146.21

After plasma treatment, the *T*_g_ values of all films increased except for Film 3. The *T*_g_ value increased due to the increase in rigidity and cross-linking within the starch and starch–PVP polymer chains of the films caused by the radicals. The enhanced *T*_g_ value correlates with other properties, such as crystallinity enhancement after plasma treatment. Chakraverty et al. also reported that the *T*_g_ value of plasma-treated composites increased with the duration of plasma treatment.^61^ The decrease of *T*_g_ in Film 3P might be due to hydrogen bond breaking, which enhances the flexibility of the cross-linked composite during plasma treatment.^62^

#### 3.2.6 Tensile Strengths of the Films

The tensile strength of the films was measured to determine their mechanical properties.^63^ Mechanical properties of films are required to understand the workability and applicability of the prepared films.^64^ In Figure 8, the tensile strength of the rice starch film is 2.68 MPa. The film’s tensile strength increased from 2.68 to 3.27 MPa after adding PVP with starch (1:1) in Film 2. Although the tensile strength of the composite Film 3 (starch and PVP–3:1) was expected to be higher than that of starch Film 1, surprisingly, it was lower than that of Film 1, which might be due to the inhomogeneity of starch and PVP in the film structure. In addition, the incorporation of Ag nanoparticles into the films decreased the tensile strength significantly. The mechanical properties of nanocomposite films depend on the interfacial interaction between nanoparticles and the biopolymer matrix, as well as the homogeneous dispersion of nanoparticles in the biopolymer matrix.^65^ In the presence of Ag nanoparticles, the degree of crystallinity increases, and tensile strength decreases, which can be correlated with the crystallinity data in Figure 8.^66^ Among Films 4, 5, and 6, Film 5 showed the highest tensile strength (0.61 MPa), as it was prepared with 1:1 starch and PVP with Ag nanoparticles. The tensile strength of Films 4 and 6 is 0.48 and 0.47 MPa, respectively. The reason behind the significant loss of mechanical strength in Ag nanoparticle-containing films is high crystallinity, uneven distribution of nanoparticles, and reduction of bond strength due to interference in the bond chain of the polymer composite.^67,68^

**Figure 8:**
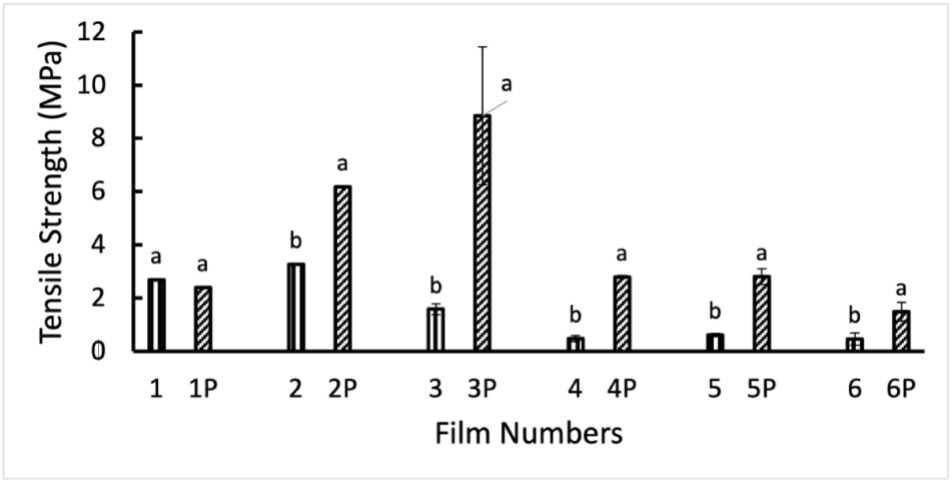
Mechanical strength of films: Film 1: starch, Film 2: starch–PVP (1:1), Film 3: starch–PVP (3:1), Film 4: starch–Ag, Film 5: starch–PVP (1:1)–Ag, Film 6: starch–PVP (3:1)–Ag. “P” denotes plasma treatment of the film.

The representative force vs time curve of Film 3 is given in Figures S19a,b for before and after plasma treatment, respectively. The tensile strength of the films significantly increased after cold plasma treatment, except for Film 1. Plasma treatment decreased the tensile strength of the starch film from 2.68 to 2.39 MPa; however, it increased the tensile strength of the 1:1 starch–PVP film from 3.27 to 6.17 MPa. The highest tensile strength was shown by the plasma-treated starch–PVP (3:1) film. This is because of the hydrogen bonding between PVP and starch in the composite film, as well as the plasma-induced cross-linking within and/or among polymers.^69^ Though Ag nanoparticle incorporation decreased the tensile strength of the films, plasma treatment significantly improved it. Overall, PVP enhances the mechanical properties of the films; Ag nanoparticles reduce them, and plasma treatment further increases the mechanical properties of the films.

#### 3.2.7 Moisture Content, Moisture Absorption, and Swelling Properties of Films

Swelling properties and water absorbance are key factors for blood and wound extrudate absorption, as well as drug diffusion and release.^70^ Moisture content and swelling properties of the films are shown in Table 3. The moisture content of all films was analyzed under 50% humidity conditions. The moisture content is higher in PVP–starch composite films than in 100% rice starch films. Moreover, the moisture content is higher in the plasma-treated films, except for Film 6P. The swelling properties of all films also increased after the addition of PVP to starch and surface treatment by cold plasma. Ag-nanoparticle-incorporated films showed lower swelling properties than control films (Table 3). These properties correlate with contact angle (CA) data, as the CA was higher in Ag-nanoparticle-containing films. One of the reasons for the increased swelling properties after plasma treatment is the incorporation of polar functional groups on the surface of the film.^71^

**Table 3:** Moisture Content and Swelling Properties of Films^a^ ^,^ ^b^

		Moisture Content ± SE		Swelling % ± SE	
Film Types	Film Numbers	Before Plasma	After Plasma	Before Plasma	After Plasma
RS	1	16.00 ± 0.0^c^	18.68 ± 0.8^b^	62.25 ± 2.1^e^	109.41 ± 0.71^b^
RS–PVP (1:1)	2	17.01 ± 0.3^b^	19.78 ± 0.2^ab^	72.11 ± 2.4^d^	113.62 ± 1.49^a^
RS–PVP (3:1)	3	19.28 ± 0.1^a^	20.17 ± 1.0^a^	64.65 ± 0.6^e^	106.85 ± 1.52^c^
RS–Ag	4	13.18 ± 0.09^d^	12.47 ± 0.0.1^c^	83.03 ± 1.2^c^	88.91 ± 1.41^d^
RS–PVP(1:1)–Ag	5	18.27 ± 0.39^ab^	22.53 ± 1.52^a^	105.54 ± 1.01^a^	106.63 ± 0.73^c^
RS–PVP(3:1)–Ag	6	13.82 ± 0.28^d^	9.27 ± 0.80^d^	102.71 ± 0.39^b^	108.64 ± 0.48^b^

a RS = rice starch.

b Values are given as mean ± SE. Different lowercase letters in the same column indicate significant differences (*p* < 0.05).

The moisture absorption capacity of the films is shown in Figure 9.^29^ Like the moisture content of the films, the moisture absorbance increased after plasma treatment. One of the reasons for this is that plasma treatment enhances the hydrophilicity of the surface, as proven by the contact angle measurements. This is due to water diffusion into the surface-treated film and the relaxation of the polymer chains, which increases the free volume.^72^ Previous studies have also reported an increase in moisture absorbance following plasma treatment.^72,73^ However, the moisture absorption capacity decreased with the addition of Ag nanoparticles to the films; however, it later increased with plasma treatment. The higher hydrophilicity of plasma-treated films compared to untreated films was attributed to the presence of oxygen-containing polar and hydrophilic groups (e.g., −OH, −CO, and −COOH) on the film surface.^50^

**Figure 9:**
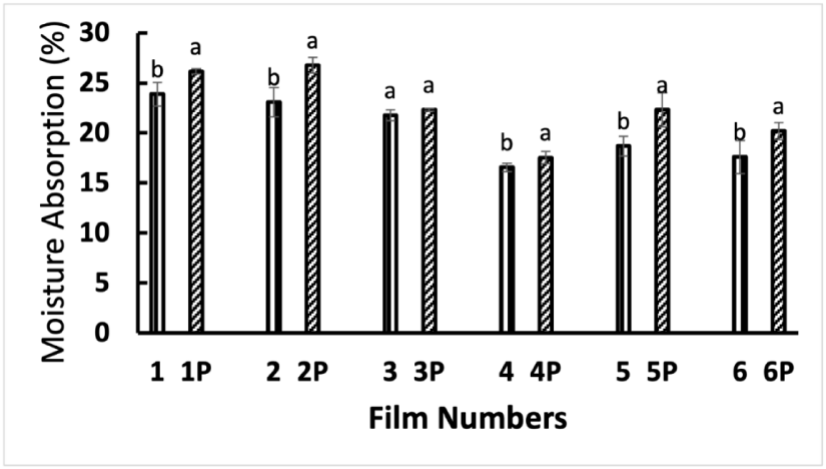
Moisture absorption of the films: Film 1: starch Film 2: starch–PVP (1:1), Film 3: starch–PVP (3:1), Film 4: starch–Ag, Film 5: starch–PVP (1:1)–Ag Film 6: starch–PVP (3:1)–Ag. “P” denotes plasma treatment of the film.

#### 3.2.8 Scanning Electron Microscopy (SEM)

Surface morphology plays a vital role in explaining changes in surface wettability. Plasma treatment can induce remarkable changes in surface morphology due to ion bombardment on the film surface and ions and radicals adsorption by the film’s surface molecules.^74,75^ In this study, the effects of plasma surface treatment on films were studied by scanning electron microscopy (SEM), and the images are shown in Figure 10. Rice starch and plasma-treated rice starch films are shown in Figure 10a,b, respectively. The surface of the rice starch film exhibits small and narrow hills and valleys due to surface etching caused by plasma treatment and chemical reactions. The increase in surface roughness also increases surface energy, which decreases the contact angle and hydrophobicity. One reason for this is the chemical etching that occurs when the sample is exposed to plasma treatment.^76^ After Ag nanoparticles are added to a starch film, the surface morphology changes as the particles act as a filler, and some parts of the film appear rough (Figure 10c). Moreover, after the surface treatment of an Ag nanoparticle-incorporated starch film by plasma, surface cleaning was observed in specific portions, while the rest of the film exhibited significant roughness and etching (Figure 10d). Moreover, the cross-sections of the starch film and the Ag-incorporated film are shown in Figure 10e,f, respectively, which represent the thickness changes as well as the inner morphology of the films after the addition of Ag nanoparticles.

**Figure 10:**
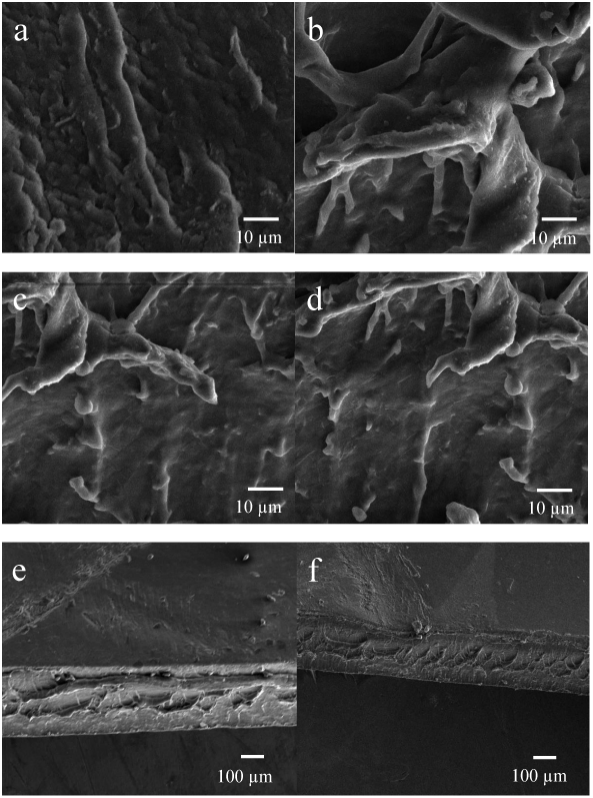
Scanning electron micrographs of the films: (a) 100% starch film, (b) plasma-treated starch film, (c) 100% starch–Ag film, (d) plasma-treated 100% starch–Ag film, (e) cross-section of 100% starch film, (f) cross-section of 100% starch–Ag film.

### 3.3 Antibacterial Activity of Films

The antibacterial activities of polymers were measured by the disk diffusion method by measuring the zone of inhibition.^77^ The zones of inhibition (ZOI) against *S. aureus* and *E. coli* were calculated after 24 and 48 h. The antibacterial activity of the films after 48 h is summarized in Table 4. Rice starch film (Film 1) showed no antibacterial activity against Gram-positive bacteria, even after plasma treatment. Rice starch–PVP (1:1) also remained inactive against Gram-positive bacteria (*S. aureus*). A ZOI of 0.15 mm was observed for the starch–PVP (3:1) composite film, which increased to 0.3 mm for the plasma-treated film (Film 3P). The plasma-treated film’s rough and oxidized surface contributes to its antibacterial properties.^78^ However, in the case of Gram-negative bacteria, only Film 2 showed a ZOI of 3.65 mm. After plasma treatment, all three films (Films 1, 2, and 3) showed antibacterial activity against Gram-negative bacteria. Film 2 (rice starch–PVP, 1:1) showed a ZOI of 4.15 mm against Gram-negative bacteria (*E. coli*).

**Table 4:** Antibacterial Properties of Films^a^ ^,^ ^b^

		Bacterial growth inhibition, 48 h, mm ± SE				
		Before Plasma Treatment			After Plasma treatment	
Film Types	Film Number	Gram +	Gram -	Film Number	Gram +	Gram -
NC		0.00	0.00		0.00	0.00
PC		6.05 ± 0.15^a^	7.35 ± 0.15^a^		6.05 ± 0.15^a^	7.35 ± 0.15^a^
RS	1	0.00 ± 0.00^a^	0.00 ± 0.15^b^	1P	0.00 ± 0.00^a^	1.85 ± 0.05^a^
RS–PVP(1:1)	2	0.00 ± 0.00^a^	3.65 ± 0.05^b^	2P	0.00 ± 0.00^a^	4.15 ± 0.05^a^
RS–PVP(3:1)	3	0.15 ± 0.07^b^	0.00 ± 0.25^b^	3P	0.30 ± 0.14^a^	2.50 ± 0.10^a^
RS–Ag	4	0.00 ± 0.00^a^	0.00 ± 0.05^b^	4P	0.00 ± 0.00^a^	1.90 ± 0.10^a^
RS–PVP(1:1)-Ag	5	0.80 ± 0.14^a^	1.00 ± 0.10^b^	5P	0.40 ± 0.1^b^	3.20 ± 0.10^a^
RS–PVP(3:1)–Ag	6	2.85 ± 0.35^a^	5.55 ± 0.25^a^	6P	2.80 ± 0.14^a^	5.80 ± 0.10^a^

a NC = negative control, PC = positive control, RS = rice starch.

b Values are given as mean ± SE. Different lowercase letters in the same column indicate significant differences (*p* < 0.05).

Antibacterial activity was significantly enhanced after the addition of Ag nanoparticles. Although Ag nanoparticles embedded in a rice starch film (Film 4) exhibited no antibacterial activity, they demonstrated antibacterial activity against Gram-negative bacteria after plasma treatment. Plasma-treated starch–PVP composite film (Film 5) showed better activity than the control film, and a ZOI of 3.20 mm was found for Gram-negative bacteria in the case of the plasma-treated film. Among all films, plasma-treated Ag-incorporated starch–PVP (3:1) played the best role in inhibiting Gram-negative bacterial growth, with a ZOI of 5.80 mm. Overall, films containing Ag nanoparticles exhibited higher antibacterial activity against Gram-negative bacteria than against Gram-positive bacteria. Gram-negative bacteria have a thinner peptidoglycan layer than Gram-positive bacteria, which allows Ag nanoparticles to penetrate the cell wall lipopolysaccharides. The cell membrane’s negative charge promotes Ag nanoparticle adhesion, allowing for easier penetration of silver ions.^19,79^ Moreover, plasma-treated films are more resistant to bacteria than any other film. Plasma-treated films increase surface roughness, which forms oxidized Ag nanoparticles that inhibit bacterial growth.^80^

The previous literature, as shown in Table 5, also demonstrated a stronger effect on Gram-negative bacteria compared to Gram-positive bacteria. However, the antibacterial activity varied among different studies, depending on the type of starch and binding material used, even though all studies had Ag nanoparticles in common.

**Table 5:** Literature Review of Antimicrobial Activity of Starch–Ag Nanoparticle Films Against Gram + and Gram– Bacteria

Substrate	ZOI (*E. coli*)	ZOI (*S. aureus)*	References
PVA–Ag films	10 mm	10.5 mm	Usman et al., (2016)^81^
Starch water–Ag np–tannic acid films	2.2 mm	3.0 mm	Jarensungnen et al., (2023)^82^
Starch–Ag/PU–nanocomposite	14.2 mm	14.1 mm	Kamali et al., (2022)^83^
Sugar palm starch–Ag np films	0.5 mm	0.5 mm	Rozilah et al., (2020)^84^
Potato starch–Ag nanocomposites	2.3 μg/mL MIC	2.2 μg/mL MIC	Taheri et al., (2014)^85^

### 3.4 Principal Component Analysis (PCA)

The multivariate analysis using principal components was independently performed for films with and without Ag nanoparticles to understand the covariance of plasma on film properties. The PCA analysis confirmed that film composition accounted for 98% of the total data variance in both cases. The starch–PVP films’ PCA showed that, while the starch (RS) film remained independent of all properties, the RS–PVP (3:1) film significantly influenced the Gram-positive antimicrobial activity and moisture absorption (Figure S21). Similarly, the RS–PVP film significantly affected the swelling index, Gram-negative antimicrobial activity, and contact angle of the films. This implies that, while an increase in PVP concentration in the film composition predominantly affected the functional properties, an excessive rise effectively caused moisture absorption and inhibited the growth of Gram-positive microorganisms. Furthermore, the biplot also clarifies that, while plasma treatment significantly altered the moisture absorption and Gram-positive microbial inhibition activities, the other properties remained unaffected in correlation with the film composition.

For films with Ag particles, the RS–Ag films affected only the contact angle, regardless of whether plasma treatments were applied (Figure S22). Similarly, RS–PVP (1:1) with Ag particles correlated with moisture absorption, and the 3:1 film significantly affected the antimicrobial properties of both Gram-positive and Gram-negative bacteria. However, the swelling index of the films was not affected by the composition of any of the three films. Additionally, only the contact angle of the films was significantly affected by the plasma treatments, while other properties remained unchanged.

### 3.5 Correlation Heatmap

The correlation heatmap illustrates the positive and negative correlations among the functional properties in relation to plasma exposure (Figure 11). The contact angle exhibits a perfect negative correlation with antimicrobial activity and the swelling index, indicating that the contact angle decreases with increasing swelling due to changes in surface properties. Similarly, all the antimicrobial activities correlate perfectly with each other, reiterating that plasma treatment has a significant effect on microbial inhibition. Additionally, it can be observed that there is very mild to no correlation between the contact angle and moisture absorption in both treated and untreated conditions. Moisture absorption shows a negative correlation of −1.0 with the antimicrobial properties after plasma treatment and ranges from −0.6 to −0.8 before treatment. These correlations indicate that these properties are interdependent and are significantly altered by plasma exposure, regardless of the film composition.

**Figure 11:**
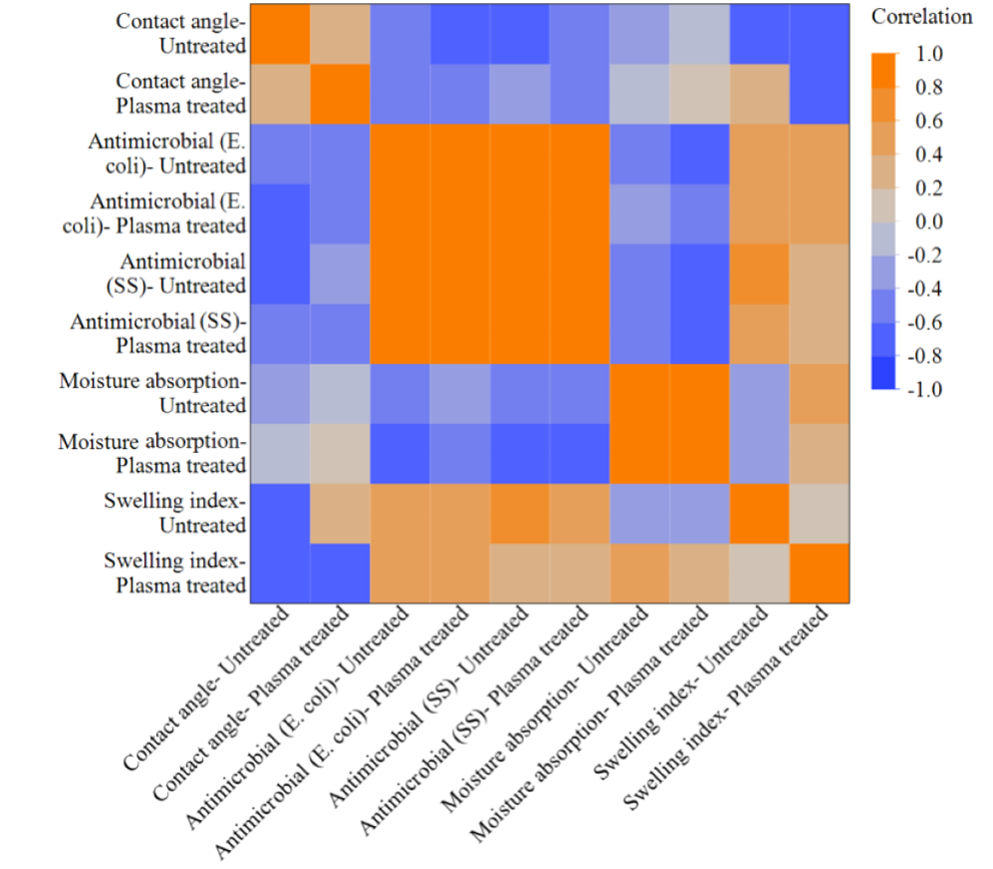
Correlation heat map of the film properties with and without plasma exposure.

In summary, plasma treatment resulted in a significant increase in crystallinity across all films, enhancing tensile strength by 10% to 80% in all samples, except for the starch film. The starch–PVP (3:1) film demonstrated the highest tensile strength increase at 80% compared with the starch film. The contact angle of all films decreased by at least 10°, indicating improved surface wettability and surface etching due to plasma exposure. Additionally, plasma treatment caused a significant increase in *T*_g_, ranging from a minimum of 3.6 °C to a maximum of 25.9 °C, with starch–PVP (1:1) film showing the highest *T*_g_ value increase of 25.9 °C after plasma treatment. These modifications were accompanied by an increase in moisture retention of approximately 5% to 18%. Regarding swelling behavior, films without Ag nanoparticles exhibited a higher swelling index, while the starch–PVP–Ag films showed a lower difference in swelling, around 10%.

## 4 Conclusion

This study demonstrates the successful synthesis of silver nanoparticles by the chemical reduction method and the development of rice starch and rice starch–PVP composite films, both with and without silver nanoparticles. The fabricated rice starch–PVP composite films have higher moisture content and swelling properties compared to the rice starch film. Surface wettability, tensile strength, thermal stability, and crystallinity were also found to be higher in the rice starch–PVP (1:1) composite film, with a few exceptions observed in the rice starch–PVP (3:1) composite film. Not only were good physical properties demonstrated, but antibacterial activity was also shown fairly in rice starch–PVP (3:1) composite films against both Gram-positive and Gram-negative bacteria, although the rice starch–PVP (1:1) film remained inactive against both *S. aureus* and *E. coli*. The Ag nanoparticle-incorporated rice starch film showed the highest contact angle, 135.14° (highly hydrophobic in nature), and the hydrophobicity decreased in starch–PVP–Ag composite films. These films’ moisture content and water absorption capacity were lower than those of films without Ag nanoparticles. Moreover, the degree of crystallinity increased significantly in Ag nanoparticle-incorporated rice starch films and starch–PVP–Ag composite films. Therefore, mechanical strength was observed to be lower in the Ag nanoparticle-incorporated rice starch film and Ag nanoparticle-incorporated rice starch–PVP composite films. The highest thermal stability was observed in the rice starch–PVP (1:1)–Ag composite film, with 41.06% residue remaining after thermal degradation at 400 °C. Though Ag nanoparticle-possessing rice starch films showed no zones of inhibition, starch–PVP (1:1)–Ag nanoparticles and starch–PVP (3:1)–Ag nanoparticles showed zones of inhibition of 0.80 mm and 2.85 mm, respectively, against Gram-positive bacteria, and 1.00 and 5.55 mm against Gram-negative bacteria after 48 h of incubation. The surface of all films was treated with cold plasma for 15 min by maintaining a distance of 5 mm from the film to the nozzle. After cold plasma treatment, crystallinity, thermal stability, glass transition temperature, and hydrophilicity increased. Though the rice starch film was not bioactive, it showed antibacterial properties after plasma treatment. The zone of inhibition of the films increased after plasma treatment against bacteria, and it was observed that the zone of inhibition against Gram-negative bacteria was higher than that against Gram-positive bacteria. Moreover, the plasma-treated rice starch–PVP (3:1)–Ag composite film showed higher activity (ZOI 5.80 mm) against Gram-negative bacteria than any other film. Thus, the present investigation explores novel insights into plasma-treated rice starch–PVP–Ag nanoparticle composite films, which is a sustainable and scalable process for antibacterial film fabrication. Ultimately, our findings could pave the way for nanocomposite films to be used in antibacterial food packaging and medical applications.

## Supplementary Material



## Notes

Color, FTIR and DLS of Ag nanoparticles, films ingredient composition, FTIR of all films, XRD and TGA comparison of all other films before and after plasma treatment, contact angle and correlation curve of all films (PDF)
