# Supplementary material for: Fabrication of Antibacterial and Functional Films from Starch-Polyvinylpyrrolidone Composite Using Plasma Treatment and Silver Nanoparticles
Source: ACS Omega. 2025 Aug 13;10(33):37419–31. doi: 10.1021/acsomega.5c03293 (PMC12391959; doi:10.1021/acsomega.5c03293)
Supplement: Supplementary file 1 [file ao5c03293_si_001.pdf]

Fabrication of Antibacterial and Functional Films from Starch-Polyvinylpyrrolidone Composite using Plasma Treatment and Silver Nanoparticles

*Muhammad Nazrul Islam, Nikitha Modupalli, Md Mahfuzur Rahman**

Department of Food Science, University of Arkansas, Fayetteville, Arkansas 72704, USA

*Corresponding Author: mahfuz@uark.edu

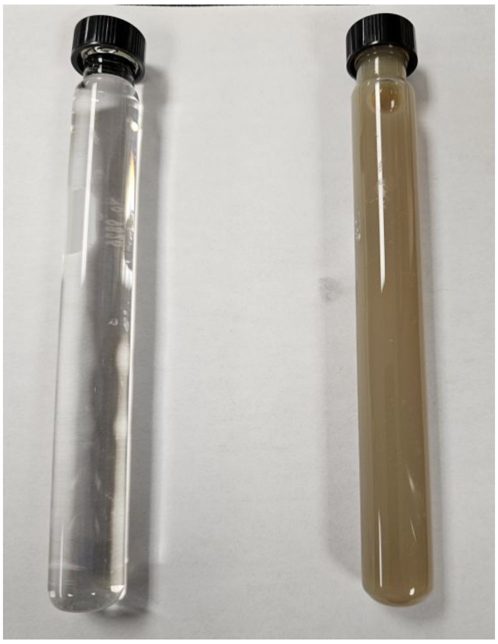

Figure S1. Color change during silver nanoparticle synthesis-a) color of silver nitrate solution b) color of silver nanoparticle

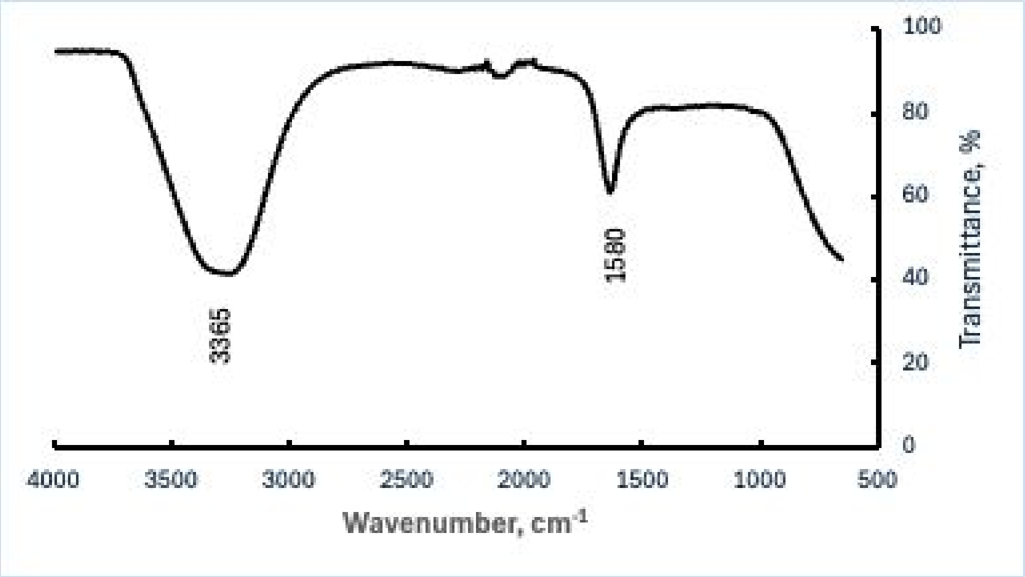

Figure S2. FTIR of Ag Nanoparticle Sol

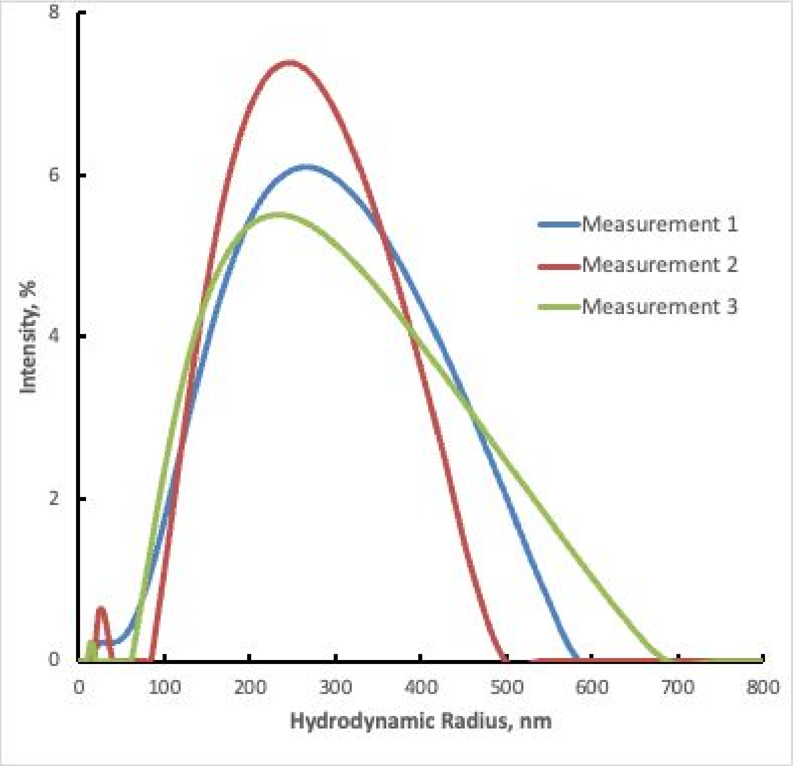

Figure S3. Dynamic light scattering (DLS) analysis of Ag nanoparticles

Table S1. Ingredients used for film formation

Film	Solvent (ml)	Starch (g)	PVP (g)	Glycerol (g)
1	DIW, 30	1.50	0.00	0.45
2	DIW, 30	0.75	0.75	0.45
3	DIW, 30	1.13	0.38	0.45
4	1% Ag Sol, 30	1.50	0.00	0.45
5	1 % Ag Sol, 30	0.75	0.75	0.45
6	1 % Ag Sol, 30	1.13	0.38	0.45

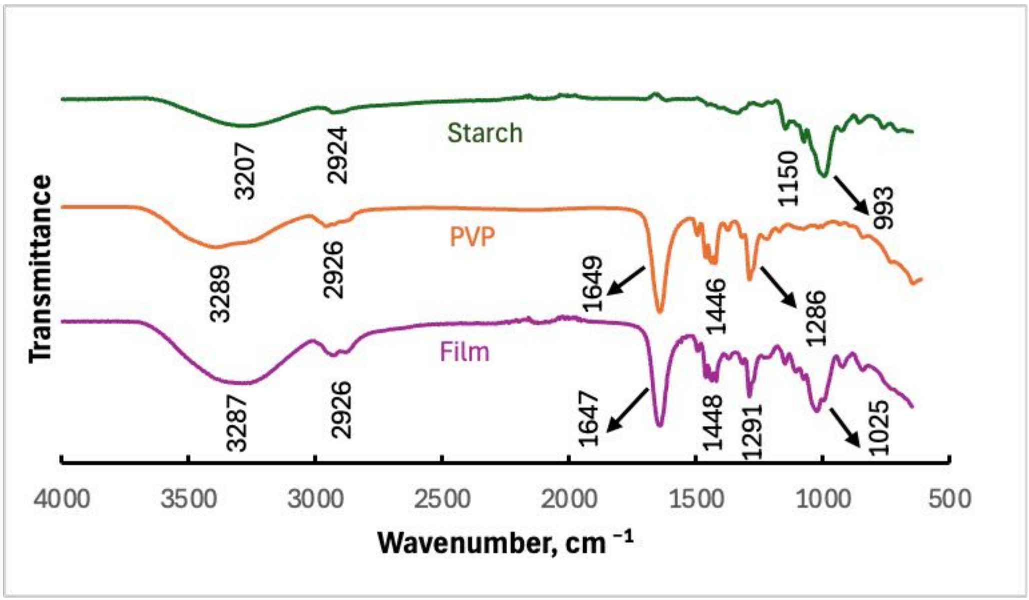

Figure S4. FTIR of starch, PVP and Starch-PVP films

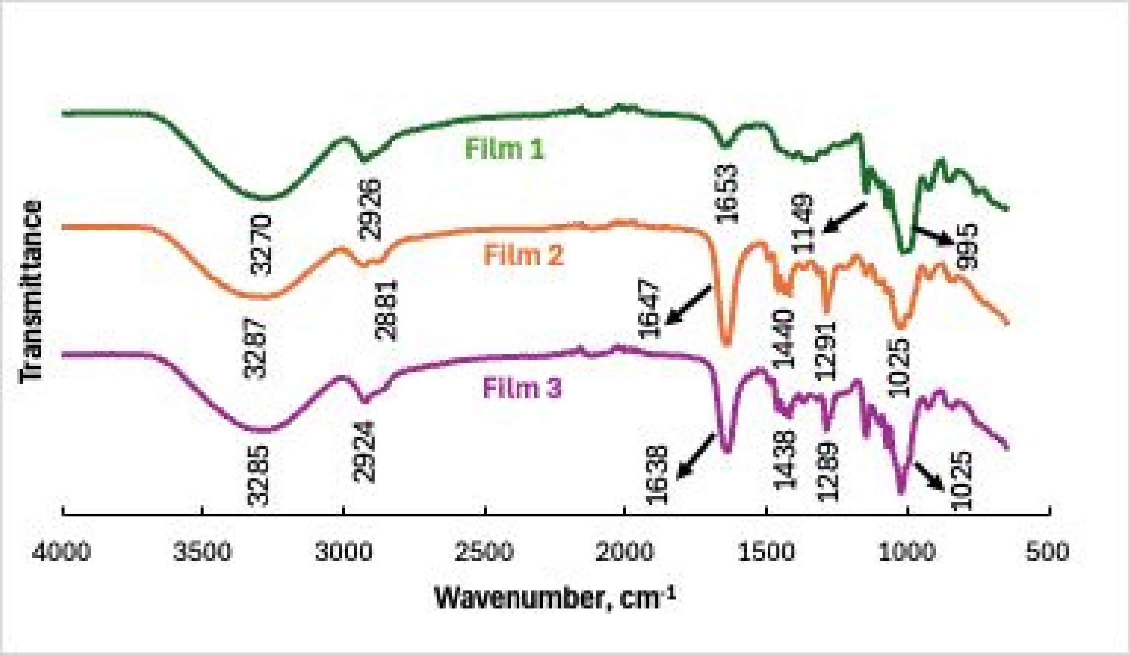

Figure S5. FTIR of films prepared by distilled water 1) 100% starch 2) 50% starch and 50% PVP and 3) 75% starch and 25% PVP

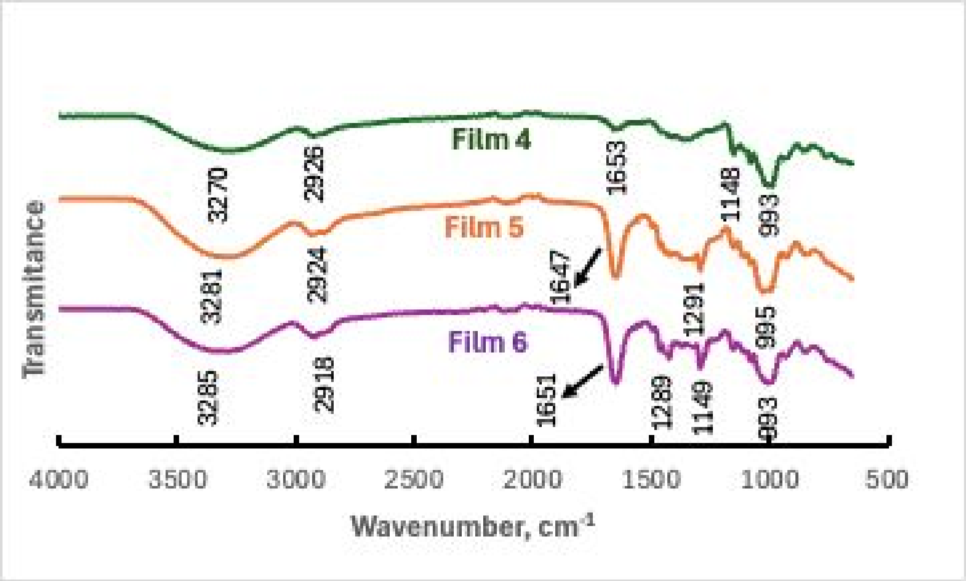

Figure S6. FTIR of films prepared by 1% silver nanoparticle sol with 4) 100% starch 5) 50% starch and 50% PVP and 6) 75% starch and 25% PVP

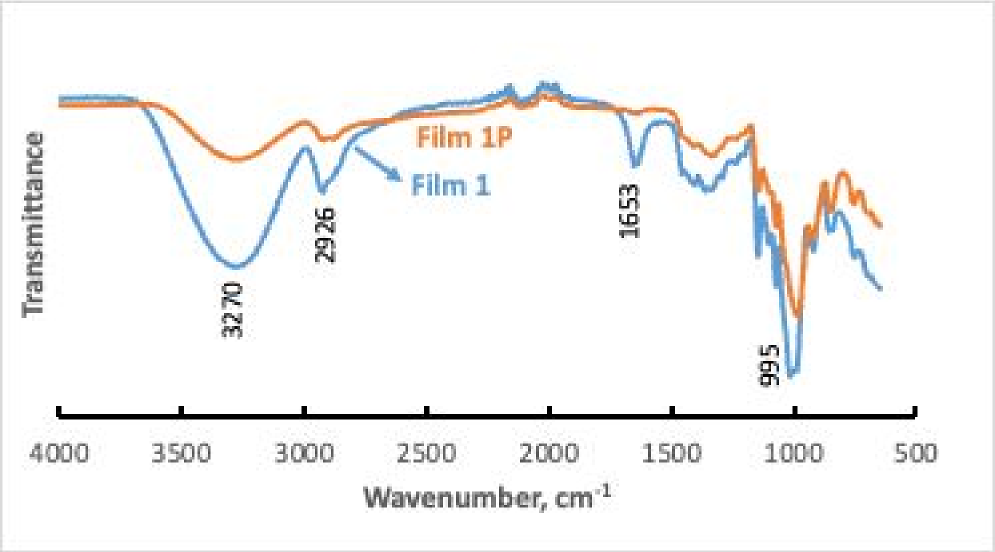

Figure S7. FTIR of film 1 prepared by 100% rice starch and film 1P treated by plasma

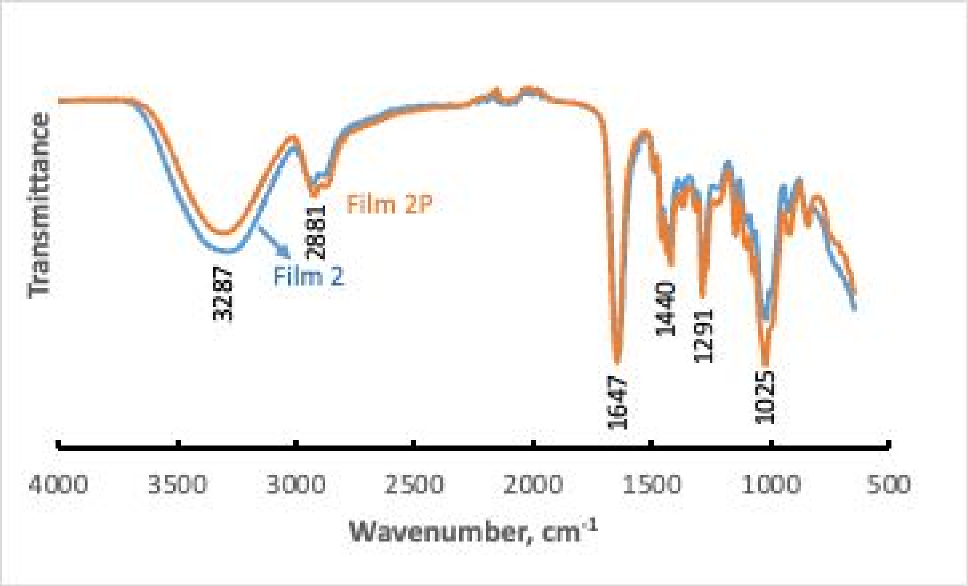

Figure S8. FTIR of film 2 prepared by rice starch and PVP (1:1) and film 2P treated by plasma

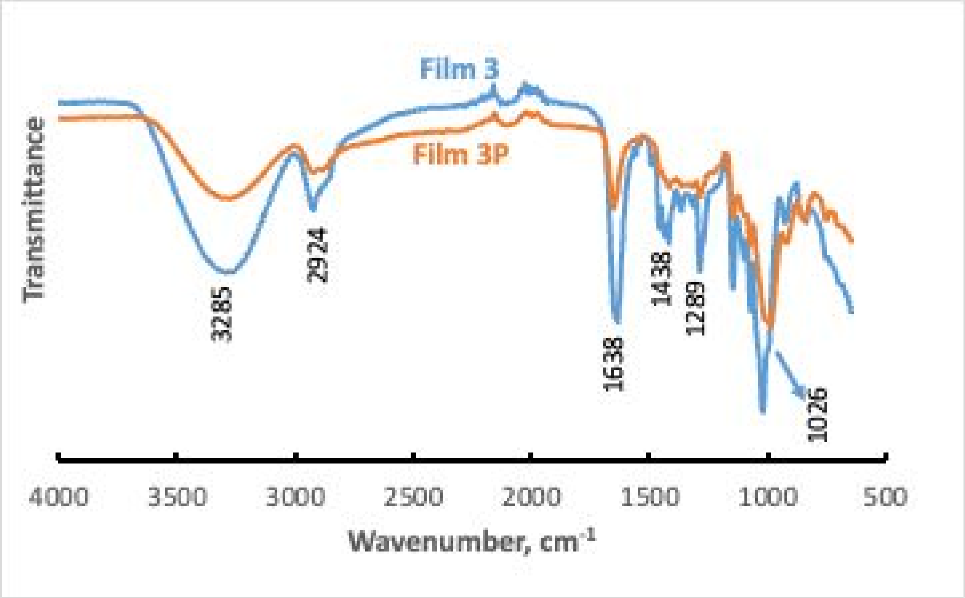

Figure S9. FTIR of film 3 prepared by rice starch and PVP (3:1) and film 3P treated by plasma

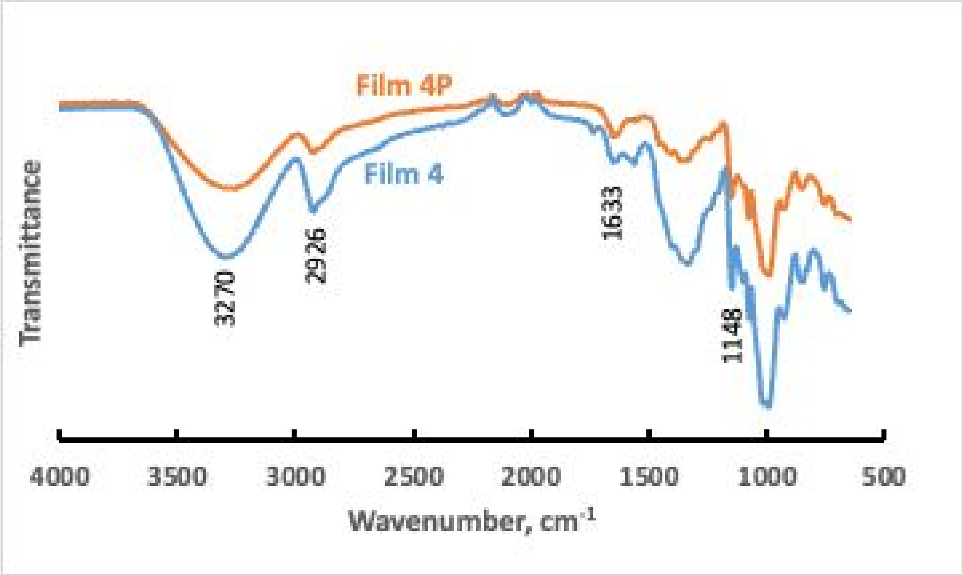

Figure S10. FTIR of film 4 prepared by 1% silver nanoparticle sol with 100% starch and 4P treated by plasma

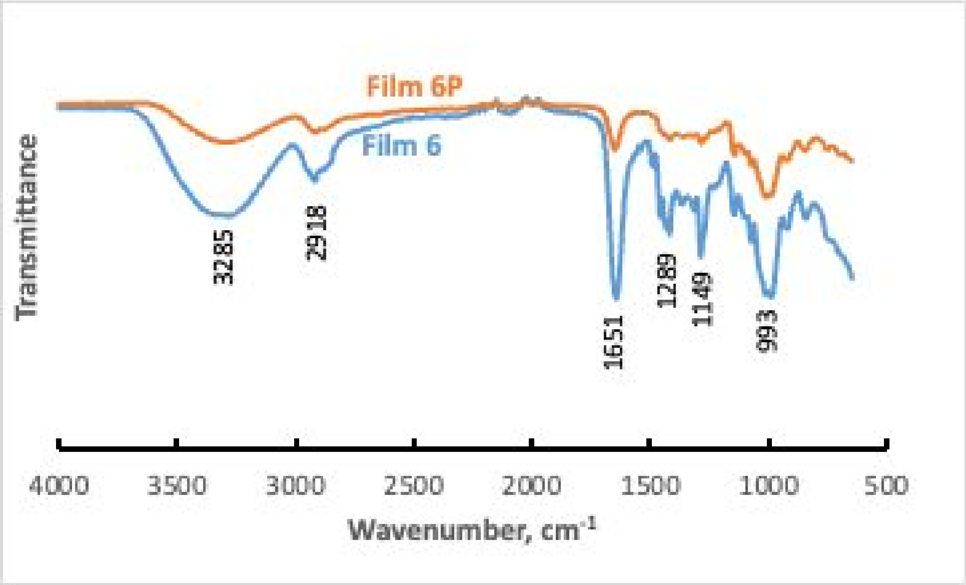

Figure S11. FTIR of film 5 prepared by 1% silver nanoparticle sol with starch and PVP (3:1) and 5P treated by plasma

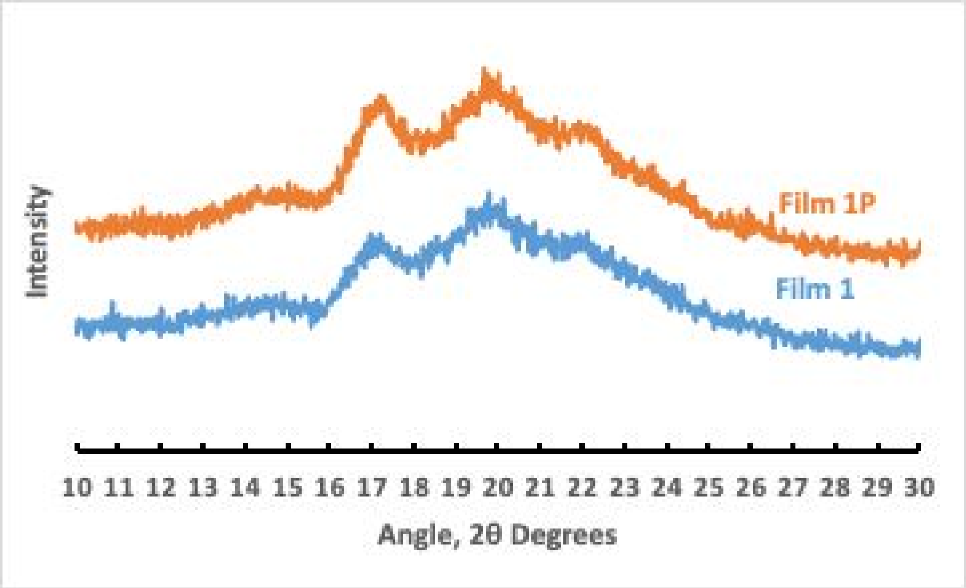

Figure S12. XRD of Rice Starch Film 1 and Fil 1P

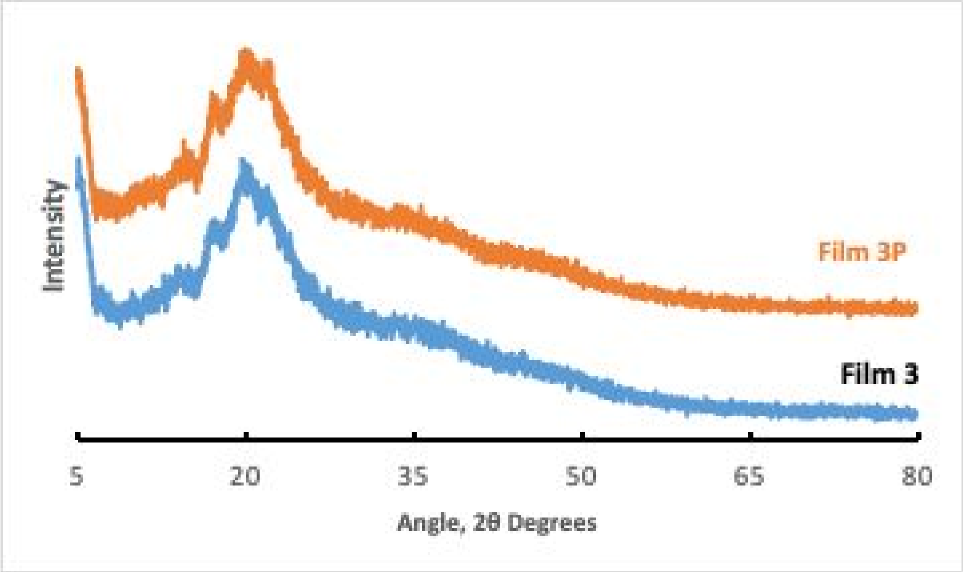

Figure S13. XRD of films – Film 3: prepared by 75% starch and 25% PVP, Film 3P: prepared by 100% starch and surface treated by plasma

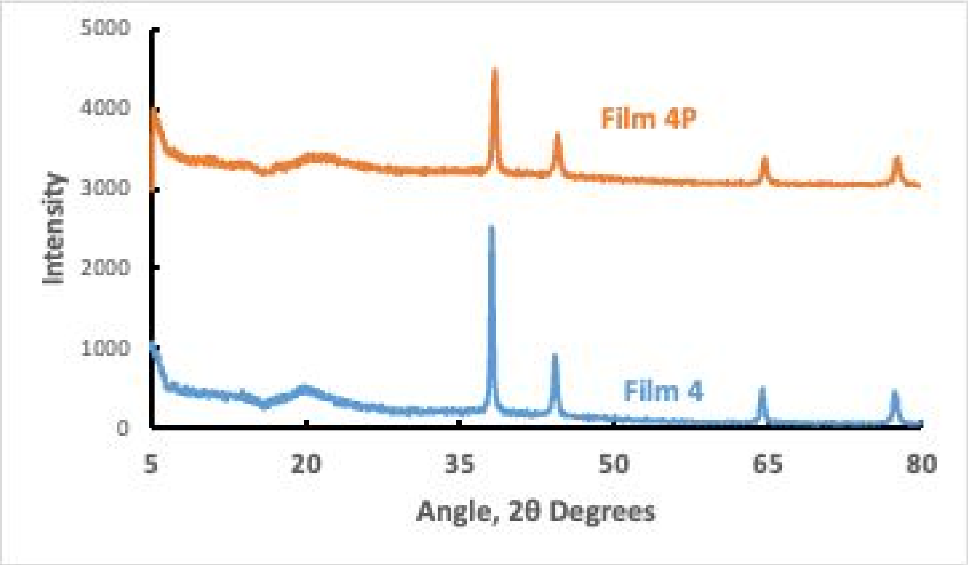

Figure S14. XRD of films – Film 4: prepared by 100% starch, Film 4P: prepared by 100% starch and surface treated by cold plasma

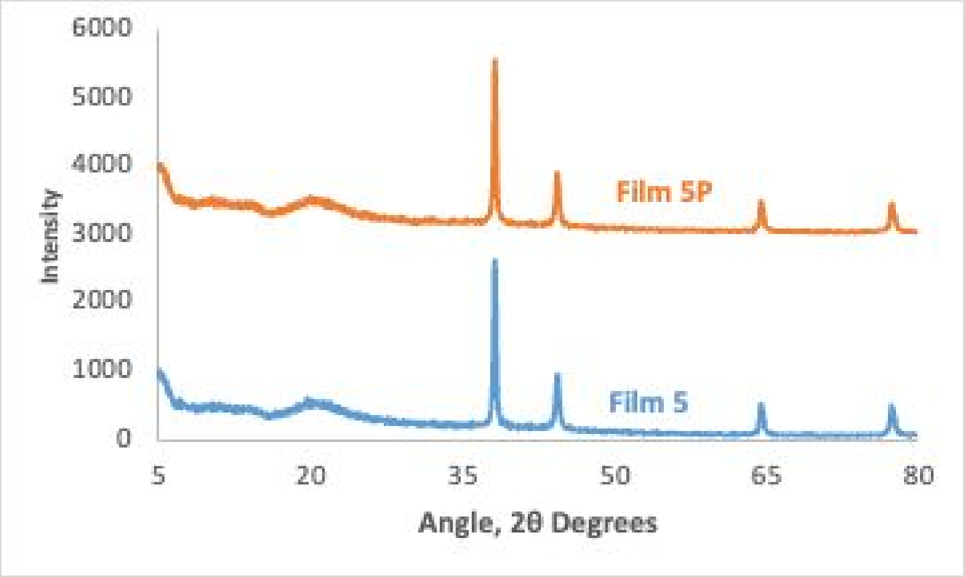

Figure S15. XRD of films – Film 4: prepared by 100% starch, Film 4P: prepared by 100% starch and surface treated by plasma

Film	Before plasma treatment	After plasma treatment
RS	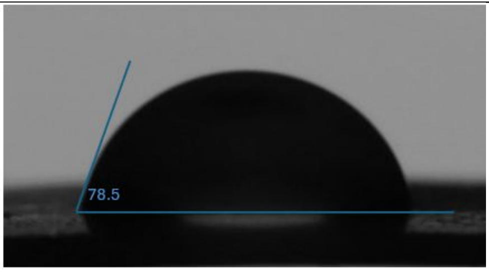	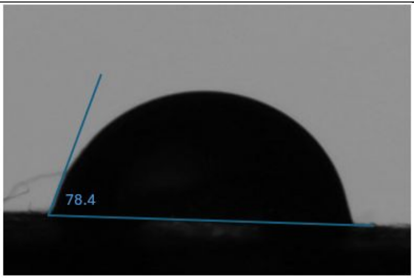
RS-PVP (1:1)	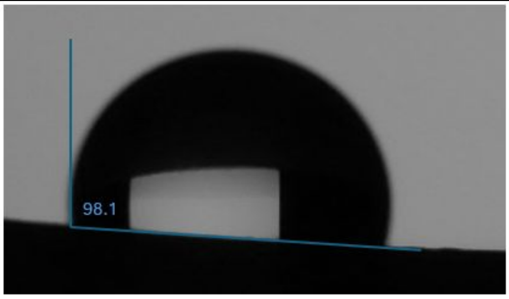	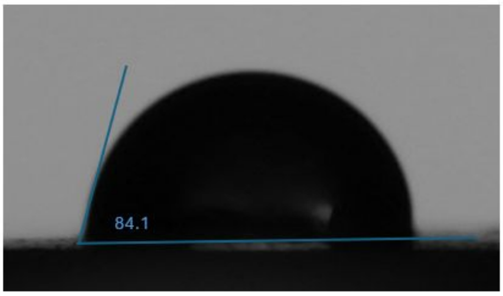
RS-PVP (3:1)	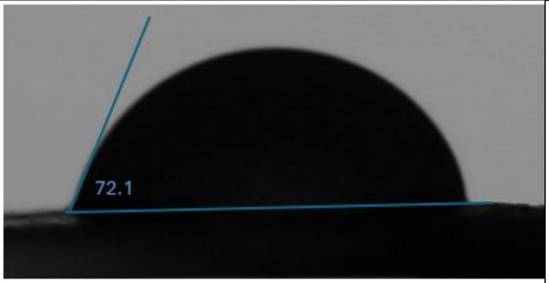	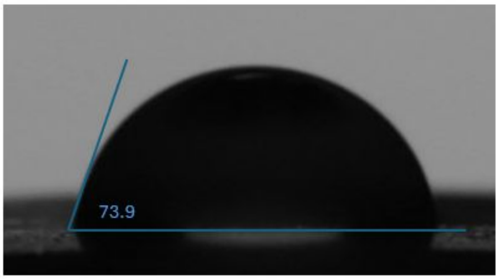
RS-Ag	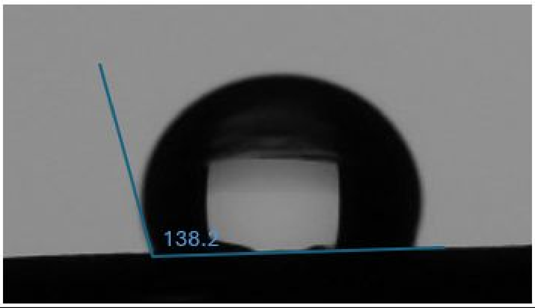	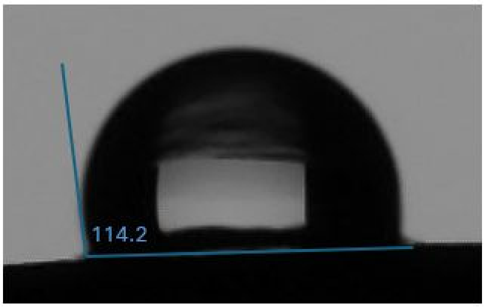
RS-PVP (1:1) - Ag	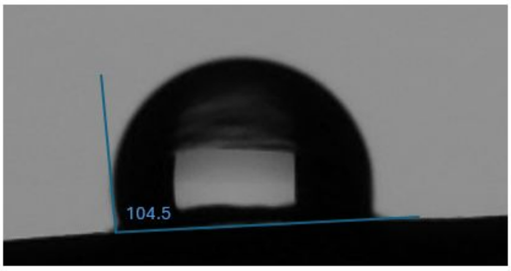	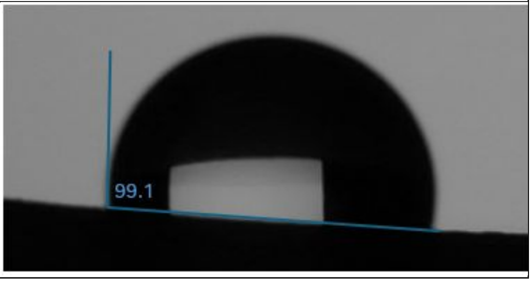

RS-PVP (3:1) - Ag	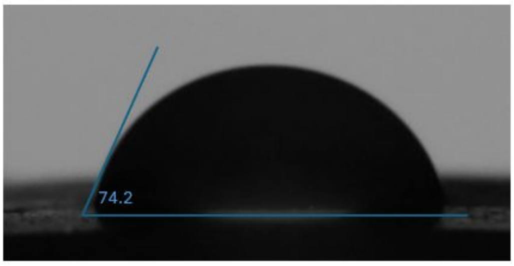	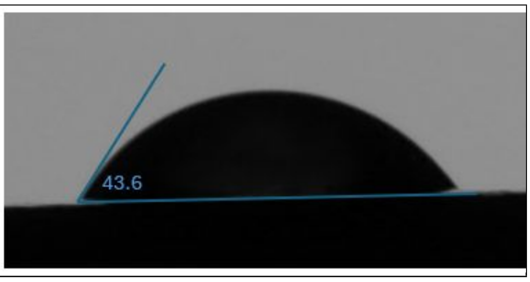

Figure S16: Contact angle of the films before and after plasma treatment

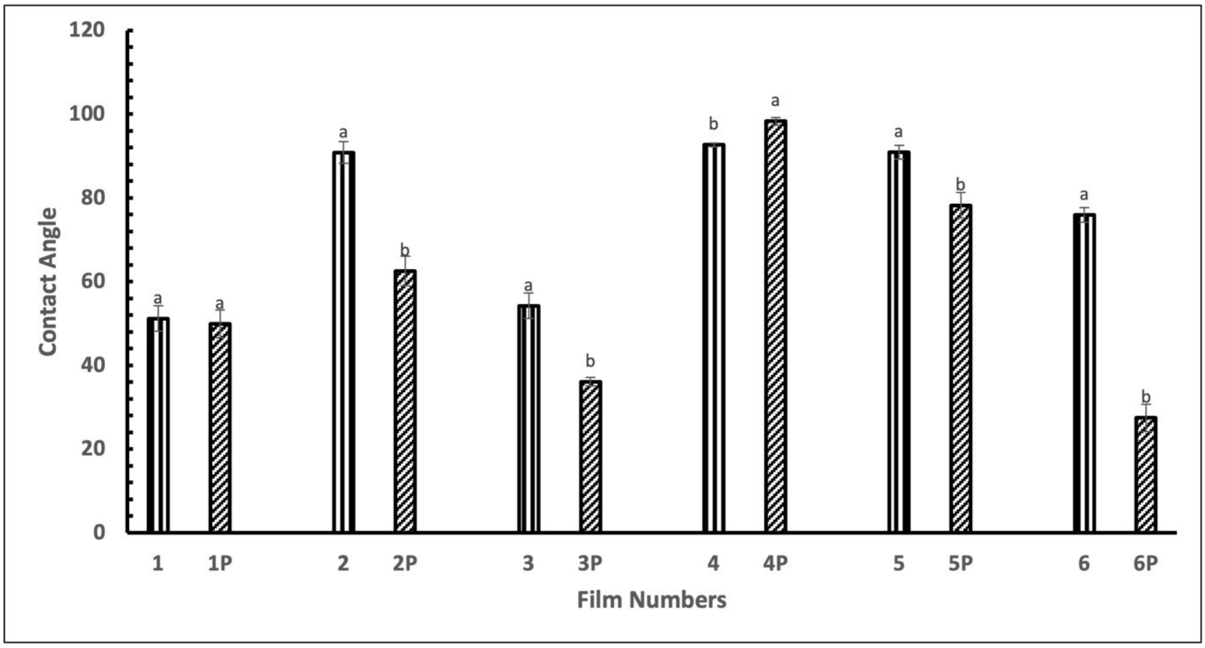

Figure S17. Contact angle of the films after 30 s

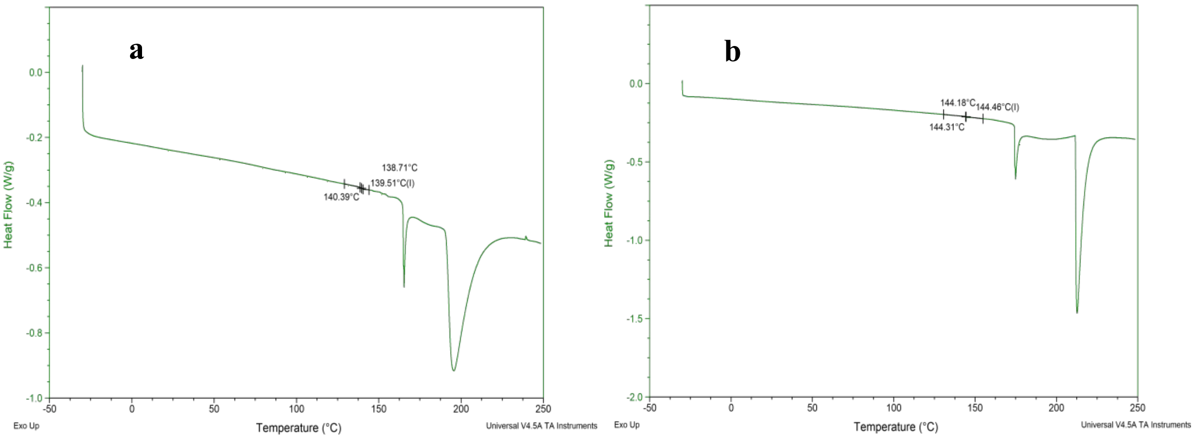

Figure S18. The Glass Transition Temperature (Tg) of starch film (film 1)- (a) before plasma treatment, and (b) after plasma treatment

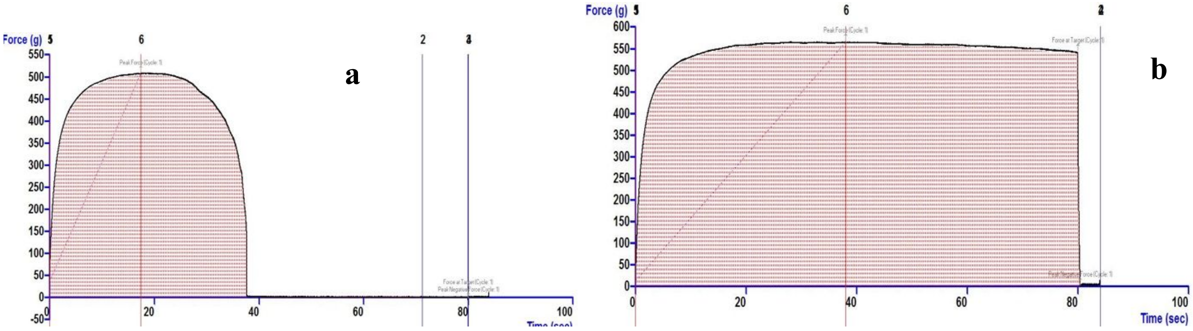

Figure S19. Force vs time curve of film 3 – (a) before plasma treatment and (b) after plasma treatment

Table S2. Bacterial growth inhibition, 24 h

Film Types	Bacterial growth inhibition, 24 h, mm±STD	
	Before Plasma Treatment	After Plasma treatment

	Film Number	Gram +	Gram -	Film Number	Gram +	Gram -
NC		0.00±0.00[c]	0.00±0.00[e]		0.00±0.00[e]	0.00±0.00[e]
PC		8.30±0.40[a]	5.65±0.05[a]		8.30±0.40[a]	5.65±0.05[a]
RS	1	0.00±0.00[c]	0.00±0.00[e]	1P	0.00±0.00[d]	2.35±0.35[d]
RS-PVP(1:1)	2	0.00±0.00[c]	4.45±0.35[c]	2P	0.00±0.00[e]	5.00±0.14[b]
RS-PVP(3:1)	3	0.15±0.07[b]	0.80±0.57[d]	3P	0.25±0.07[c]	2.65±0.64[c]
RS-Ag	4	0.00±0.00[c]	0.13 ±0.18[de]	4P	0.00±0.00[e]	2.35±0.21[d]
RS-PVP(1:1)-Ag	5	0.00±0.00[c]	0.60±0.14[d]	5P	0.05±0.07[de]	2.85±0.49[c]
RS-PVP(3:1)-Ag	6	0.15±0.07[b]	5.05±0.78[b]	6P	0.45±0.07[c]	5.85±0.07[a]

NC=negative control, PC=positive control, RS=rice starch

Table S3. Bacterial growth inhibition, 72 h

Film Types	Bacterial growth inhibition, 72h, mm±STD					
	Before Plasma Treatment			After Plasma treatment		
	Film No	Gram+	Gram -	Film No	Gram+	Gram -

NC		-1.75±0.25^{c}	-1.90±0.10^{e}		-	-
PC		5.75±0.05^{a}	7.35±0.07^{a}		-	-
RS	1	-1.25±0.05^{c}	-2.50±0.20^{e}	1P	-0.95±0.07^{d}	-1.95±0.05^{d}
RS-PVP(1:1)	2	-0.15±0.05^{c}	-3.90±0.10^{c}	2P	-0.20±0.00^{e}	-2.70±0.10^{b}
RS-PVP(3:1)	3	-0.25±0.07^{b}	-10.00±0.00^{d}	3P	0.00±0.00^{c}	-10.00±0.00^{c}
RS-Ag	4	-0.55±0.07^{c}	-0.25 ±0.15^{de}	4P	-0.35±0.05^{e}	-0.15±0.05^{d}
RS-PVP(1:1)-Ag	5	-0.95±0.05^{c}	-1.70±0.10^{d}	5P	-0.55±0.05^{de}	-0.25±0.05^{c}
RS-PVP(3:1)-Ag	6	-1.55±0.05^{b}	-4.65±0.05^{b}	6P	0.45±0.07^{c}	-1.20±0.10^{a}

NC=negative control, PC=positive control, RS=rice starch

Film	Before plasma	After plasma
RS	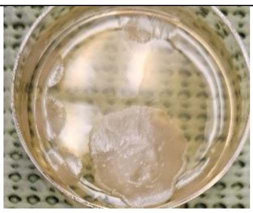	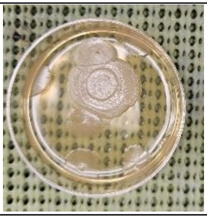

RS-PVP (1:1)	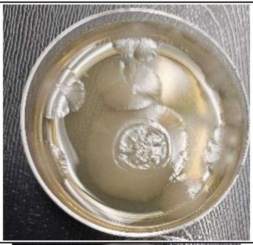	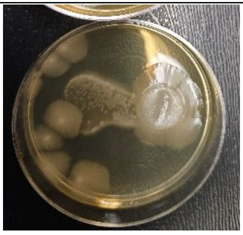
RS-Ag	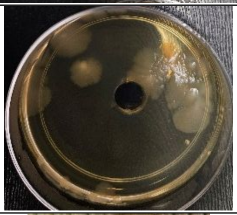	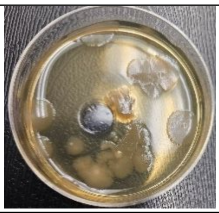
RS-PVP (1:1)- Ag	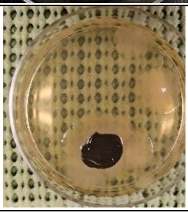	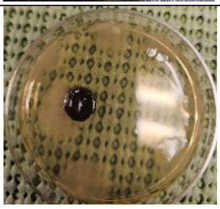
RS-PVP (3:1)- Ag	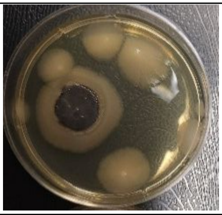	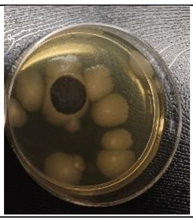

Figure S20. The antibacterial activity for the films after 72 hours

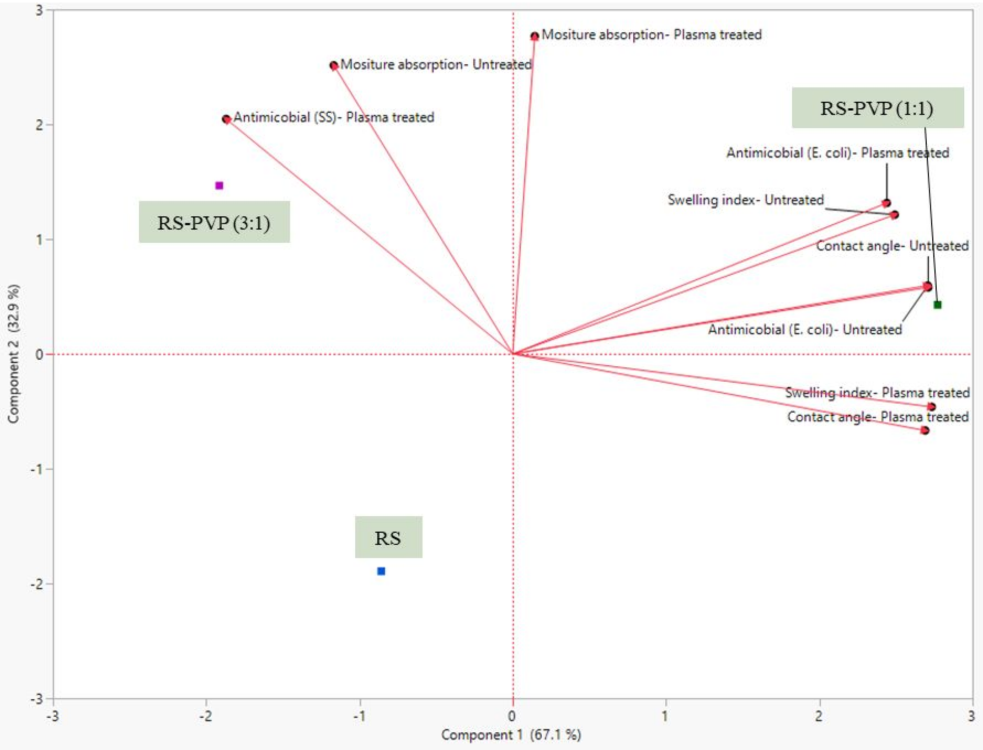

Figure S21. Principal component analysis Biplots according to correlation of starch-PVP film composition on functional properties with and without plasma treatment

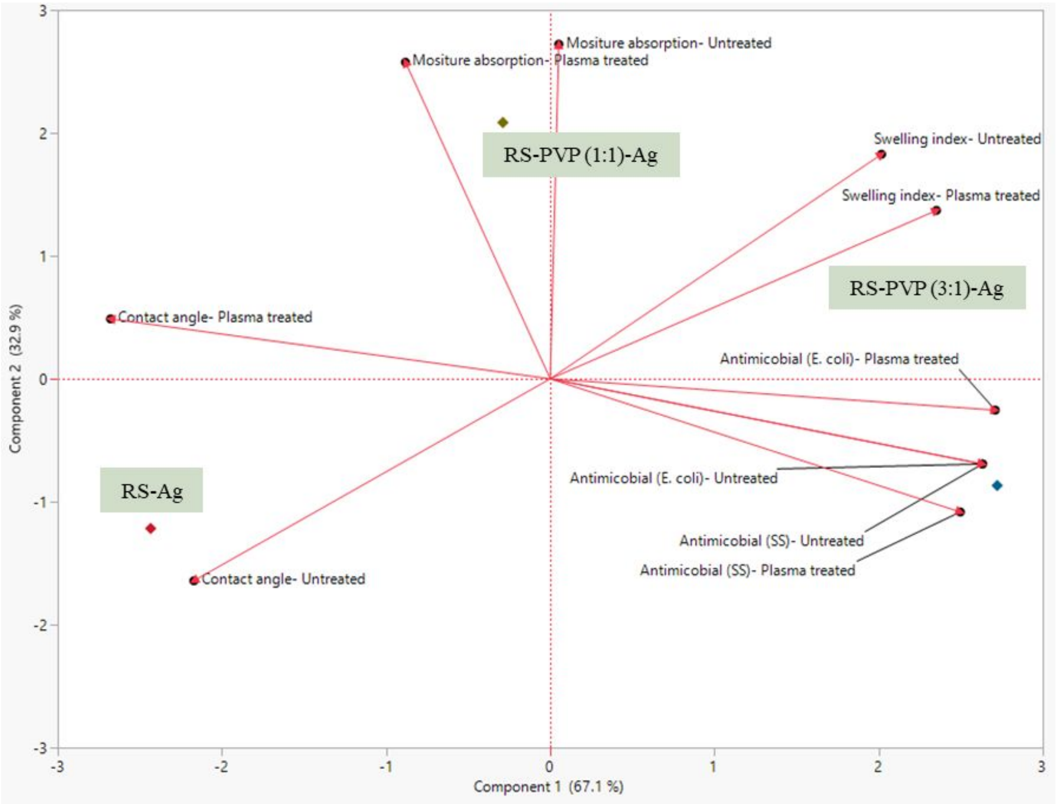

Figure S22. Principal component analysis Biplots according to correlation of Ag particles- RS film composition on functional properties with and without plasma treatment
